# Causal relationship and potential pathogenic mechanisms between rosacea with pharyngeal and laryngeal cancer

**DOI:** 10.1016/j.bjorl.2025.101636

**Published:** 2025-04-25

**Authors:** Zexin Zhu, Xiaoxue Wang

**Affiliations:** a Department of Surgical Oncology, The Comprehensive Breast Care Center, The Second Affiliated Hospital of Xi’an Jiaotong University, Xi’an, China; b Department of Dermatology, The Second Affiliated Hospital of Xi’an Jiaotong University, Xi’an, China

**Keywords:** Rosacea, Cancer, Mendelian randomization, Causal relationship, Pharyngeal and laryngeal cancer

## Abstract

**Objective:**

Rosacea is a common chronic inflammatory skin disease. Observational studies reported the association between rosacea and cancer. The causal relationship between rosacea and cancer has been reported rarely.

**Methods:**

We employed a two-sample Mendelian Randomization (MR) study to assess the causality between rosacea and 17 subtypes of cancers. Rosacea and cancers’ association Genome-Wide Association Study (GWAS) data were collected. The Inverse Variance Weighted (IVW) method was utilized as the principal method in our Mendelian Randomization (MR) study, with additional using the MR-Egger, weighted median, simple mode, and weighted mode methods. The MR-Egger intercept test, Cochran’s *Q* test, MR-Pleiotropy RESidual Sum and Outlier (MR-PRESSO) and leave-one-out analysis were conducted to identify heterogeneity and pleiotropy. After that, Disease-related genes were also referenced from GeneCard.

**Results:**

After our MR analysis, we found rosacea has a causal relationship with pharyngeal and laryngeal cancer (PLC, Odds Ratio [OR = 1.137], 95% CI 1.010‒1.283, *p*-value = 0.035). Rosacea may increase the risk of PLC. No causal link between rosacea and other cancer was observed. No heterogeneity or pleiotropy was observed. Reversed MR showed PLC has no causal effect on rosacea. Furthermore, disease-related genes were obtained. The intersecting genes between rosacea and PLC were identified. Gene Ontology (GO) and Kyoto Encyclopedia of Genes and Genomes (KEGG) enrichment analyses were performed, these intersecting genes were associated with pathways related to immunity inflammation procedures or pathways.

**Conclusion:**

This study provided new evidence of the relationship between rosacea and PLC. Rosacea may play a facilitative role in PLC progression. Identification of related genes related to rosacea and PLC offers new scene for investigating the potential pathogenesis of PLC, this could provide innovative and efficient ways to address PLC treatment.

**Level of evidence:**

Level II. Mendelian Randomized (MR) studies are second only to randomized controlled trials in terms of the level of evidence.

## Highlights

• A variety of MR analysis methods are used.
• Causal link between Rosacea and PLC (Pharyngeal and Laryngeal Cancer).
• No causal relationship between Rosacea and other cancer.
• Sensitivity analysis confirm the robustness and reliability of the findings.
• Explored the molecular pathogenic mechanism between Rosecea and PLC.

## Introduction

Rosacea is a prevalent and chronic inflammatory skin disease, predominantly affects cheeks, chin, nose, forehead and the eyes.^1^, ^2^ Statistically, the prevalence rate of rosacea ranges from 1% to 22%, depending on geographical location and population.^3^, ^4^ Recently, a systematic review resulted the prevalence of rosacea was estimated at 5.5% of the adult population worldwide.^5^ Unlike previous studies which found a greater prevalence in women,^1^, ^2^ the result of systematic review showed both men and women are equally susceptible to the condition.^5^ The pathophysiology of rosacea remains unclear. Mechanistically, rosacea pathogenesis is relevant to different inflammatory pathways, which include dysregulation of the innate and adaptive immune system.^1^, ^6^ As suggested by the analysis of Single Nucleotide Polymorphisms (SNPs) in genes associated with rosacea, genetic factors may also play a role.^7^ Certain triggers, such as stress, ultraviolet light, spicy food, smoking, and alcohol can exacerbate the symptoms of rosacea.^2^ The diagnosis of rosacea is based on clinical features and skin-biopsy.^2^ Treatment options for rosacea include skin care, topical medications like brimonidine and ivermectin, oral drugs such as doxycycline and minocycline, and biologics like Secukinumab and Erenumab.^1^, ^8^, ^9^, ^10^ It is important to note that, rosacea is a chronic condition, although patients can have remissions based on several treatments, relapses commonly occur.^1^, ^2^

Studies also reported that rosacea is associated with many other disorders. For example, an observational study reported that rosacea is linked to a higher risk of depression and anxiety disorders, the risk grows as the disease severity increases.^11^ The risk of death due to gastrointestinal diseases significantly increased in patients with rosacea.^12^ A systematic review suggested that Inflammatory Bowel Diseases (IBDs) was bidirectionally associated with rosacea.^13^ Rosacea also has a relationship with Parkinson’s disease,^14^ and Chronic Obstructive Pulmonary Disease (COPD).^15^ Additionally, the possible relationship between rosacea and different cancers has been reported.^16^

Mendelian Randomization (MR) utilizes one or more genetic variants as Instrumental Variables (IVs) based on Genome-Wide Association Studies (GWAS). MR studies can infer the causal effects of exposure on an outcome. Recently, MR analysis also reported the causal relationship between rosacea and other disease. For instance, MR analysis showed that IBD has a cause impact on rosacea.^17^ To our knowledge, limited studies have yet investigated the causal effect of rosacea on the risk of cancer using Mendelian randomization. Our investigation aimed to explore the rosacea variants as instrumental variables for cancer risk utilizing two-sample MR.

## Methods

### Study design

**Fig. 1:**
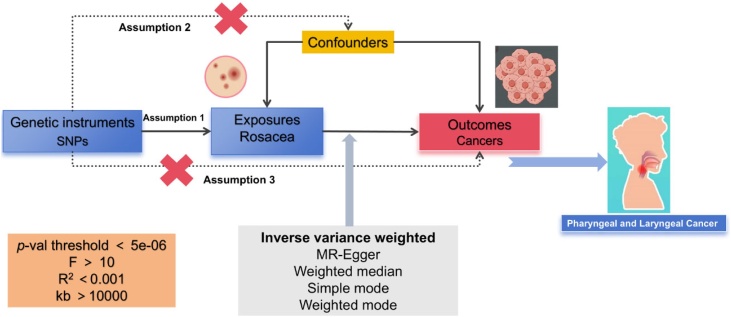
Flowchart schematic diagram followed by the MR analysis’ principal of this study.

According to the MR framework (Fig. 1), three key assumptions are included: 1) Relevance Assumption: Single Nucleotide Polymorphisms (SNPs) that are substantially linked to exposures are used as Instrumental Variables (IVs). 2) Independence Assumption: These SNPs (IVs) should not show any correlation with the relevant confounding factor. 3) Exclusivity Assumption: These SNPs (IVs) should affect outcomes only through its effect on exposure.^18^, ^19^, ^20^

### Data sources

We utilized summary data associated with rosacea and 17 subtypes of cancers from MRC Integrative Epidemiology Unit Open GWAS database (https://gwas.mrcieu.ac.uk). Accession numbers finn-b-L12_ROSACEA for rosacea, 1,195 cases and 211,139 controls, information of different subtypes of cancers were detailed in Table 1, summary data were accessed from IEU Open GWAS project database (https://gwas.mrcieu.ac.uk). Our study was conducted by secondary analysis of data from other studies, all participants or their family members have provided informed written consent in the original studies.

**Table 1:** Information of GWAS summary data source included in the study.

Trait	GWAS ID	Sample size	No. of SNPs	Year
Rosacea	finn-b-L12_ROSACEA	/	16,380,452	2021
Pharyngeal and laryngeal cancer	ebi-a-GCST90018898	355,564	19,083,781	2021
Breast cancer	ebi-a-GCST90018799	257,730	24,133,589	2021
Cervical cancer	ebi-a-GCST90018817	239,158	24,138,337	2021
Endometrial cancer	ebi-a-GCST90018838	240,027	24,135,295	2021
Esophageal cancer	ebi-a-GCST90018841	476,306	24,194,380	2021
Gastric cancer	ebi-a-GCST90018849	476,116	24,188,662	2021
Hepatic bile duct cancer	ebi-a-GCST90018803	476,091	24,196,592	2021
Hepatic cancer	ebi-a-GCST90018858	475,638	24,194,938	2021
Lung cancer	ebi-a-GCST90018875	492,803	24,188,684	2021
Ovarian cancer	ebi-a-GCST90018888	246,520	24,137,758	2021
Pancreatic cancer	ebi-a-GCST90018893	476,245	24,195,229	2021
Prostate cancer	ebi-a-GCST90018905	211,227	24,119,306	2021
Skin cancer	ebi-a-GCST90018921	492,203	24,178,924	2021
Thyroid cancer	ebi-a-GCST90018929	491,974	24,198,226	2021
Colorectal cancer	ebi-a-GCST012879	32,072	38,356,021	2018
Bladder cancer	ieu-b-4874	373,295	9,904,926	2021
Cancer of urinary tract	ukb-d-C_URINARY_TRACT	361,194	10,309,627	2018

### Instrumental variables (IVs) selection

Related IVs for MR analysis followed particular principles: SNPs should be associated with exposures at the locus-wide significance level: *p*-value < 5e-06. In addition, Linkage Disequilibrium (LD) coefficient *r^2^* should be less than 0.001, not closely related (clumping window more than 10,000 kb) to ensure exposure instrument independence. We used the F statistic to measure the strength of the IVs, the values of *F*-statistics were more than 10.^18^, ^19^, ^20^

### MR analysis

Causal associations between rosacea and cancers were determined utilization MR analysis. In the exposure-outcome analysis, we employed MR with more than two SNPs serving as IVs. Our MR analysis using each of the five methods: Inverse Variance-Weighted (IVW) was performed as the primary statistical analysis method in our MR analysis for evaluating causal effects, besides, weighted median, and MR-Egger, simple mode, weighted mode were utilized.^18^, ^19^, ^20^

The heterogeneity of the chosen SNPs was evaluated using Cochrane’s *Q* test, a *p-*value of more than 0.05 suggested the lack of heterogeneity. The random effects model was used once significant heterogeneity has been identified. We evaluated the possible bias from horizontal pleiotropy using the weighted median and MR-Egger regression in order to gauge the robustness of the IVW method. The MR-PRESSO (MR-Pleiotropy RESidual Sum and Outlier) test was used to appraise outliers that might have been influenced by horizontal pleiotropy. The causal-effect estimates for individual variants were displayed using Scatter plot. Thereafter, we performed a “leave-one-out” analysis to examine the stability of the results in the context of a single SNP’s influence and presented the findings in a forest plot.^18^, ^19^, ^20^

All statistical analysis were conducted in R software (Version 4.3.2) using the TwoSampleMR package (Version 0.5.8). The statistical significance level is *p*-value < 0.05. Pooled ORs (Odds Ratio) with 95%CI were calculated.

### Acquisition the related genes and Enrichment Analysis

Disease related genes were obtained from The GeneCards database (https://www.genecards.org/), using the key word “Rosacea” and causal related cancer (Pharyngeal and Laryngeal Cancer [PLC], as resulted). Intersection of the disease genes were used to obtain the crosstalk genes. Gene Ontology (GO) categories of Molecular Function (MF), Biological Process (BP), and Cellular Component (CC), Kyoto Encyclopedia of Genes and Genomes (KEGG) enrichment analysis were performed on the obtained crosstalk genes in rosacea and related cancer (PLC). Adjust *p*-value < 0.05 was used as a filtering condition.

## Results

### Instrumental variables

According to the quality control principle as mentioned, 13 SNPs related with rosacea were adopted as Instrumental Variables (IVs). The SNPs included in the exposure data are detailed in Supplementary Table S1.

### MR analysis

**Fig. 2:**
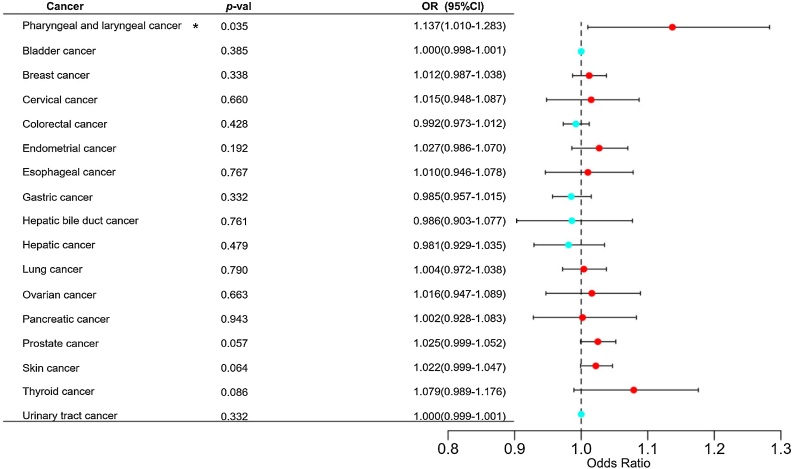
Forest plot of Mendelian randomization analysis for rosacea on 17 subtypes of cancers risk. OR, Odds Ratio; CI, Confidence Interval; *p-value < 0.05.

**Fig. 3:**
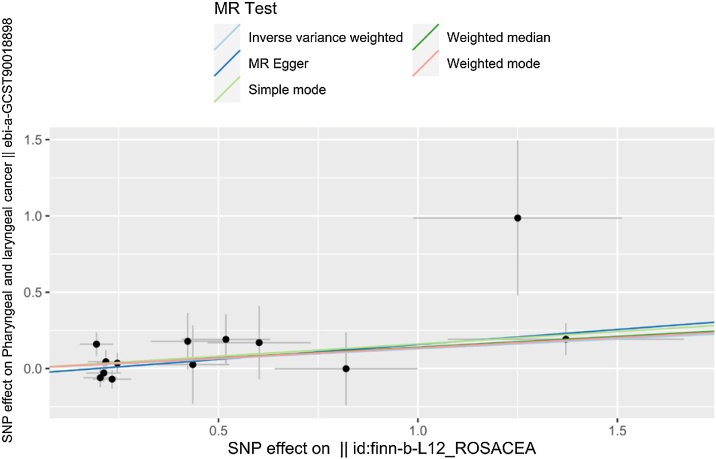
Scatter plots showing significant causal effects between rosacea and Pharyngeal and laryngeal cancer.

We conducted the two sample MR analysis between rosacea and 17 subtypes of cancers. The IVW MR analysis demonstrated that rosacea has a causal relationship on the risk of pharyngeal and laryngeal cancer (PLC, [OR = 1.137], 95% CI 1.010‒1.283, *p*-value = 0.035, Table 2). No causal association between rosacea and other cancers was observed (Fig. 2). Using the MR-Egger, the relationship between rosacea and PLC was visualized (Fig. 3).

**Table 2:** Causal relationship between Rosacea and PLC.

Exposure	Outcome	Methods	*p-*value
		Inverse variance weighted	0.035
		MR-Egger	0.055
Rosacea	Pharyngeal and laryngeal cancer	Weighted median	0.098
		Simple mode	0.331
		Weighted mode	0.105

PLC, Pharyngeal and Laryngeal Cancer.

### Sensitivity analysis

**Fig. 4:**
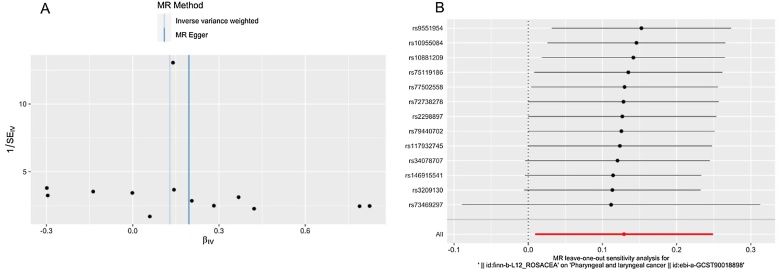
(A) Funnel plot of our Mendelian randomization study. (B) Results of “Leave-one-out” sensitivity analysis in our Mendelian randomization study.

According to the analysis of Cochran’s *Q* test, our IVW-MR analysis results demonstrated no evidence of heterogeneity among the reported results. Furthermore, the MR-Egger regression and MR-PRESSO analysis results provided evidence that there exists no significant horizontal pleiotropy in our MR analysis (Table 3). The symmetric funnel plot (Fig. 4A) indicated no evidence of horizontal pleiotropy. We also conducted leave-one-out method to identify and delete abnormal instrumental variables. The results showed the robustness of our results (Fig. 4B). These results suggest that the MR analysis results were relatively stable.

**Table 3:** Sensitivity analysis of our MR.

*Q*	*p-*value for Cochran Q test	Egger-intercept	*p-*value for MR-Egger intercept	*p-*value for MR-PRESSO Global test
12.465	0.409	−0.038	0.346	0.539

### Reversed MR

We also conducted the reversed MR. We identified 14 SNPs whose *p-*values were less than 5e-06 out of a total of 19,083,781 SNPs from PLC (detailed in Supplementary Table S2). No significant results were detected when rosacea was treated as the outcome (Table 4).

**Table 4:** Reverse causality between PCL and Rosacea.

Exposure	Outcome	Methods	*p-*value
		Inverse variance weighted	0.487
		MR-Egger	0.417
Pharyngeal and laryngeal cancer	Rosacea	Weighted median	0.402
		Simple mode	0.438
		Weighted mode	0.391

PLC, Pharyngeal and Laryngeal Cancer.

### Crosstalk genes and enrichment analysis

**Fig. 5:**
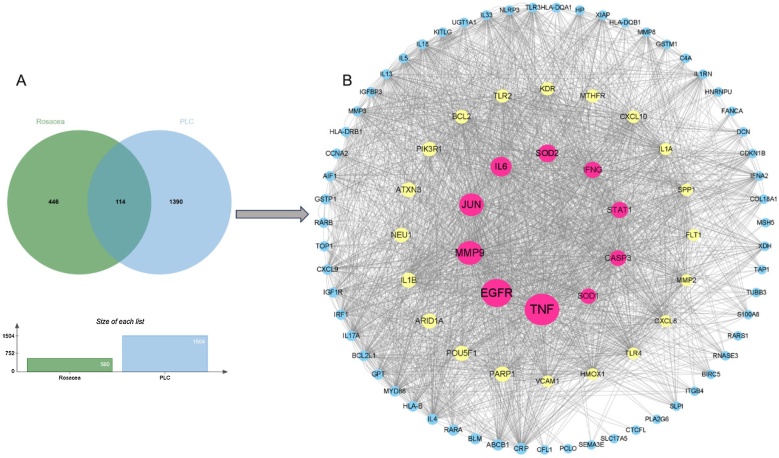
(A) Intersecting genes between rosacea and PLC; (B) PPI network of genes associated with rosacea linked to PLC risk.

A total of 560 rosacea disease genes, 1,504 PLC disease genes were obtained from the GeneCard, respectively. As shown in Fig. 5A, considering the intersection of these diseases, 114 intersecting genes were obtained (Supplementary Table S3 for details). In order to clarify the interactions between genes at the protein level, we employed STRING (https://cn.string-db.org/) to develop Protein-Protein Interaction (PPI) networks (Fig. 5B), which depict the top 10 nodes identified as hub genes (TNF, EGFR, MMP9, JUN, IL-6, SOD2, IFNG, STAT1, CASP3, SOD1).

**Fig. 6:**
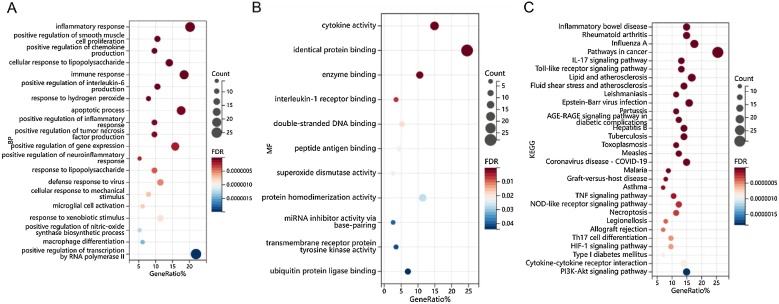
(A) Biological process (BP); (B) Molecular function (MF); (C) Kyoto Encyclopedia of Genes and Genomes (KEGG) Enrichment analysis.

Furthermore, we performed GO and KEGG (Kyoto Encyclopedia of Genes and Genomes) enrichment analysis. GO results showed that the set of genes associated with inflammatory response, immune response, positive regulation of interleukin-6 production, positive regulation of tumor necrosis factor production, defense response to virus at the biological level Biological Process (BP) analysis, they were primarily associated with cytokine activity, identical protein binding, enzyme binding, interleukin-1 receptor binding, transmembrane receptor protein tyrosine kinase activity ubiquitin protein ligase binding at the Molecular Function (MF) analysis (Fig. 6A, B). The KEGG enrichment results showed that pathways in cancer, IL-17 signaling pathway, Toll-like receptor signaling pathway, Epstein-Barr virus infection, AGE-RAGE signaling pathway in diabetic complications, TNF signaling pathway, NOD-like receptor signaling pathway, Th17 cell differentiation, HIF-1 signaling pathway, Cytokine-cytokine receptor interaction, PI3K-Akt signaling pathway were significantly enriched among the crosstalk genes of rosacea and PLC, as shown in Fig. 6C. Related proteins predominantly localized within the extracellular space, extracellular region, external side of plasma membrane, extracellular exosome, cell surface, MHC class II protein complex nuclear chromosome, endosome membrane and so on (Fig. S1, Table S4 for details).

## Discussion

We conducted a MR analysis to investigate the causal relationship between rosacea and 18 subtypes of cancers. Our results showed that rosacea has a causal impact on PLC, in contrast, PLC has no causal effect on rosacea. To the best of our knowledge, the causal relationship between rosacea and cancers has rarely reported.

Neoplasms of the pharynx rank as the 7^th^ most common type of cancer and the 9^th^ leading cause of cancer-related deaths globally.^21^ Accordingly, there are approximately 710,000 new cases and 359,000 fatalities each year.^21^ The main risk factors for pharyngeal cancers include tobacco use, alcohol consumption, and betel quid consumption.^21^, ^22^ Infection with Human Papillomavirus (HPV) and Epstein-Barr Virus (EBV) is also a known risk factor.^21^ Our KEGG enrichment results also showed that Epstein-Barr virus infection may involve in rosacea related PLC (Fig. 6C). Laryngeal cancer is considered to be a relatively rare malignant neoplasm, accounting for only 0.8% of all new cancer cases each year, in 2015, it was estimated that there were 13,560 new cases of laryngeal cancer and 3,640 deaths from this disease (in the United States).^23^ The incidence of laryngeal cancer tends to increase with age and is more prevalent in men than in women. Likewise, factors associated with the development of laryngeal cancer are smoking and alcohol consumption.^23^ Similarly, cancers of the pharynx and larynx are treated using a combination of chemotherapeutic, radiation, and surgical techniques, specific approach depends on various factors such as the type, biology, location, and stage of the cancer, as well as the patient’s individual circumstances and other considerations.^24^, ^25^

As mentioned, observational studies have reported the relationship between rosacea and cancers,^16^, ^26^, ^27^, ^28^ meanwhile, different studies have produced varying results. A nationwide cohort study of the Danish population reported an increased risk of hepatic cancer, Non-Melanoma Skin Cancer (NMSC),^26^ and breast cancer among patients with rosacea, and the risk of incident lung cancer was significantly decreased in patients with rosacea; while a study in China reported a higher incidence of breast cancer and glioma, but a lower prevalence of hematological cancer in patients with rosacea.^27^ According to a systematic review, rosacea patients were more likely to suffer from non-melanoma skin cancers, breast cancer, and glioma, there was no significant link found between rosacea and melanoma, the associations between rosacea and hepatic or thyroid cancers were unclear due to conflicting results.^29^ Intriguingly, another MR reported a causal link between rosacea and HER-negative malignant neoplasm of the breast risk.^30^ However, our MR study did not find the causal relationship between rosacea and skin cancer, breast cancer, or hepatic cancer. Rosacea-associated chronic inflammation may exhibit immune-suppressive microenvironments, potentially attenuating the pro-carcinogenic effects of systemic inflammation. Prior studies rarely differentiate different subtypes of rosacea, which have distinct inflammatory profiles. For example, papulopustular rosacea (Th1/Th17-driven)^31^ may correlate more strongly with cancers linked to chronic IL-17 exposure (related to PLC). The role of rosacea-related inflammation may vary depending on the tumor’s immune landscape. We will propose subtype-specific analyses to reconcile these discrepancies.

To our known, no studies reported the relationship between rosacea and PLC, our MR analysis uncovered new evidence suggesting the potential link. Chronic immune dysregulation associated with rosacea may be an explanation for how rosacea could increase the risk of PLC. Immune dysregulation plays a fundamental role in the development of rosacea, with activation of the innate immune system leading to heightened cytokine production.^32^, ^33^ Individuals with rosacea have higher levels of LL-37 (cathelicidin antimicrobial peptide) expression in their skin, which is then processed into shorter fragments. The increased presence of LL-37 promotes the degranulation and release of inflammatory mediators.^32^, ^33^, ^34^ Shorter fragments of LL-37 have the properties of antimicrobial and immune-activation, play a role in promoting angiogenesis, inducing leukocyte chemotaxis, and contributing to the production of proinflammatory cytokines.^35^ Generally, neoplastic growth is associated with chronic infections, inflammation, tissue injury, and tissue regeneration, involve LL-37 antibacterial and immunomodulatory functions. This link suggests that the LL-37 peptide may play a role in the development of cancer. An increasing amount of evidence indicated that LL-37 can exhibit dual and conflicting impacts, either promoting or inhibiting tumor growth.^36^ Overexpression of LL-37 was found to enhance development and progression of ovarian, lung cancer.^37^ Additionally, LL-37 has been identified as a potential growth factor for malignant melanoma.^38^ Meanwhile, the mechanism of how rosacea increased PLC risk remains uncertain. Our functional enrichment analysis showed several interleukin and Tumor Necrosis Factor (TNF) played roles in rosacea and PLC, immune and inflammation pathways may also contribute to the onset and progression of PLC. For instance, IL-17 is a well-known inflammatory mediator that plays important roles in pathogenesis of inflammatory skin diseases. Previous studies reported that Th17 pathway is activated in rosacea and IL-17, one of Th17 signature cytokines, is elevated in tissue samples of rosacea patients.^39^ IL-17 is a highly versatile pro-inflammatory cytokine crucial for a variety of processes, including host defense, tissue repair, the pathogenesis of inflammatory disease and the progression of cancer. chronic IL-17 activity orchestrates pathogenic responses that promote cancer and autoimmunity. A growing body of evidence strongly support a pathogenic role for IL-17 in carcinoma formation, including cancers of the colon, skin, pancreas, liver, lung and myeloma, also PLC.^40^, ^41^ Analogously, TNF-α is also a commonly reported biomarker in rosacea,^42^ may promote proliferation, survival, migration, and angiogenesis of chemotherapy-resistant cancer cells, thus facilitating tumor development. TNF-α exerts its effects through two receptors, TNFR1 and TNFR2, and its role in the tumor microenvironment is complex and context-dependent.^43^

There are several limitations to our study. First, due to the original GWAS statistics, we were unable to divide the cohorts or perform subgroup analyses. Second, our analysis only included individuals of the European population. Although using a single European population to investigate causal relationships can minimize population stratification bias, it is important to interpret these findings with caution regarding their applicability to other populations. The Odds Ratio (OR) of 1.137 reflects a modest magnitude of association. While given the high prevalence of rosacea (estimated 5%‒10% in adults), 13.7% increase in PLC risk per unit increase in genetic predisposition to rosacea could translate to substantial attributable risk at the population level. Our finding resulted rosacea has nominal causal connections with PLC, but these correlations vanished after applying the Bonferroni correction. *p-*value above the Bonferroni-corrected threshold, but lower than 0.05 considered suggestive evidence for a potential causal association. It is important to note that the Bonferroni correction can result in false negatives.^44^ The reverse MR analysis indicated no causal relationship between PLC and rosacea. However, confounders such as HPV infection, which is increasing globally, might affect this association. Additionally, other potential confounders could also impact the study results. We have explicitly acknowledged that this study does not provide direct technical guidance for surgical decision-making. The current evidence should be interpreted as complementary to clinical risk assessment tools. Further research is required on the mechanism of how rosacea increased PLC risk.

## Conclusions

In conclusion, our MR study has provided the first-ever evidence that rosacea has a causal impact on PLC. Which indicated rosacea increased the risk of PLC. Our results provided a potential connection between rosacea and PLC. Rosacea may be a novel avoidable strategy in the treatment of PLC.

## CRediT authorship contribution statement

All authors have read and approved the submission of the manuscript. Xiaoxue Wang presented research questions. Xiaoxue Wang and Zexin Zhu contributed to the data retrieval, statistical analysis, and visualization of results. Xiaoxue Wang and Zexin Zhu conducted a thorough critical review and revised the manuscript before its submission.

## Ethics approval and consent to participate

The study used the large publicly available GWAS database, which has received approval from their relevant ethical review board and participants.

## Funding

This work has been funded by the 10.13039/501100001809National Natural Science Foundation of China (82404921).

## Data availability

No original data were generated in the present study. The datasets mentioned in this article are publicly available. https://gwas.mrcieu.ac.uk. Details see Supplementary Tables.

## Declaration of competing interest

The authors declare no conflicts of interest.

## Footnotes

Appendix A Supplementary material related to this article can be found, in the online version, at doi:https://doi.org/10.1016/j.bjorl.2025.101636.
