# Supplementary material for: Causal relationship and potential pathogenic mechanisms between rosacea with pharyngeal and laryngeal cancer
Source: Braz J Otorhinolaryngol. 2025 Apr 25;91(4):101636. doi: 10.1016/j.bjorl.2025.101636 (PMC12056395; doi:10.1016/j.bjorl.2025.101636)

**BJORL-D-24-00467_ Supplementary materials**

**Table Supplementary S1** Information of SNPs instrumental variants associated Rosacea.

| **SNP** | **chr** | **pos** | **beta** | **se** | **p-val** | **effect_allele** | **other_allele** | **eaf** |
| --- | --- | --- | --- | --- | --- | --- | --- | --- |
| rs146915541 | 1 | 247271819 | 1.2502 | 0.2617 | 1.79E-06 | A | G | 0.008133 |
| rs2298897 | 1 | 29165837 | 0.2176 | 0.0456 | 1.78E-06 | G | C | 0.2933 |
| rs10881209 | 1 | 105447612 | -0.2124 | 0.0438 | 1.23E-06 | C | T | 0.6544 |
| rs79440702 | 2 | 18930611 | 0.6019 | 0.1299 | 3.58E-06 | T | C | 0.02903 |
| rs34078707 | 5 | 119111988 | 0.5186 | 0.1112 | 3.11E-06 | G | T | 0.03905 |
| rs72738278 | 5 | 16137633 | -0.2466 | 0.0534 | 3.89E-06 | C | T | 0.1896 |
| rs75119186 | 6 | 70205134 | 0.8199 | 0.1791 | 4.68E-06 | A | G | 0.01548 |
| rs10955084 | 8 | 97825208 | 0.2037 | 0.0421 | 1.29E-06 | T | C | 0.427 |
| rs77502558 | 11 | 97829352 | 0.4357 | 0.0918 | 2.09E-06 | T | C | 0.05716 |
| rs9551954 | 13 | 31278440 | 0.2333 | 0.0483 | 1.39E-06 | C | T | 0.7526 |
| rs73469297 | 15 | 96820417 | 1.3709 | 0.296 | 3.64E-06 | T | C | 0.006435 |
| rs117932745 | 17 | 22001624 | 0.4228 | 0.0921 | 4.44E-06 | A | G | 0.05661 |
| rs3209130 | 22 | 23804685 | 0.1941 | 0.0418 | 3.38E-06 | G | A | 0.4692 |

**Table Supplementary S2** Information of SNPs instrumental variants associated Pharyngeal and laryngeal cancer using for reverse MR.

| **SNP** | **chr** | **pos** | **Beta** | **se** | **p-val** | **effect_allele** | **other_allele** | **Eaf** |
| --- | --- | --- | --- | --- | --- | --- | --- | --- |
| rs186348643 | 4 | 164563662 | 1.8199 | 0.3878 | 2.69E-06 | G | T | 0.015001 |
| rs1131769 | 5 | 138857919 | -0.4169 | 0.0852 | 1.00E-06 | C | T | 0.870158 |
| rs7724367 | 5 | 29426747 | -0.4151 | 0.0827 | 5.23E-07 | A | G | 0.146488 |
| rs7719151 | 5 | 157524334 | 0.2769 | 0.0602 | 4.30E-06 | T | G | 0.64842 |
| rs197967 | 6 | 23582665 | 0.337 | 0.0731 | 4.05E-06 | T | C | 0.2959 |
| rs4263660 | 7 | 5206038 | -0.4802 | 0.1013 | 2.11E-06 | G | T | 0.915346 |
| rs2710770 | 10 | 92437631 | -0.4816 | 0.1017 | 2.20E-06 | A | C | 0.798699 |
| rs3824870 | 11 | 132081873 | 0.2978 | 0.0646 | 4.08E-06 | A | G | 0.253379 |
| rs190108525 | 11 | 23839099 | 2.6193 | 0.5651 | 3.56E-06 | A | G | 0.007996 |
| rs73105532 | 12 | 52935419 | 0.541 | 0.1049 | 2.52E-07 | A | G | 0.088677 |
| rs11063148 | 12 | 4530737 | 0.392 | 0.0854 | 4.38E-06 | T | C | 0.171407 |
| rs11619847 | 13 | 112934946 | 1.6093 | 0.3487 | 3.93E-06 | T | G | 0.020801 |
| rs7181744 | 15 | 95105481 | 0.3733 | 0.0798 | 2.88E-06 | C | T | 0.1604 |
| rs187770245 | 16 | 63014124 | 1.3599 | 0.2955 | 4.19E-06 | G | A | 0.024508 |

**Table Supplementary S3**

| Rosacea, PLC, Rosacea\|PLC |
| --- |
| CAMP, BRCA2, NEAT1 |
| IRF4, BRCA1, IL17A |
| AHR, ATM, STAT1 |
| TRPV4, TP53, GSTM1 |
| ADAMDEC1, MLH1, MMP9 |
| CTSS, CDH1, TLR2 |
| CTSZ, CHEK2, IL6 |
| TRD-GTC9-1, PMS2, IL1B |
| CETP, PTEN, S100A8 |
| VDR, C11orf65, MMP3 |
| PTGDR, NF1, MYD88 |
| AHSG, AXIN2, TRPM8 |
| S100A9, DICER1, CXCL8 |
| PWAR1, CDKN2A, IL33 |
| MIR148B, POLE, TLR3 |
| ZNF667-AS1, MET, KDR |
| TRC-GCA24-1, NBN, HP |
| MIR378G, TSC2, IL18 |
| ALPI, STK11, TNF |
| CGA, RB1, CRP |
| NISCH, RET, TLR4 |
| TRN-GTT2-1, ALK, MMP8 |
| TRN-GTT2-7, PIK3CA, VEGFA |
| ADAM9, SMAD4, TMX2-CTNND1 |
| RHOH, SMARCA4, GSTP1 |
| SMAD5-AS1, KRAS, GSTT1 |
| EMSLR, BRAF, LINC01672 |
| RNY5, ERBB2, MIR196A1 |
| CDSN, PTCH1, MIR196A2 |
| SPINK5, MSH3, MIR152 |
| GJA1, PDGFRA, MIR148A |
| USP8, MRE11, MIR296 |
| C1QB, CTNNB1, SPP1 |
| GNB2, KIT, FLT1 |
| AIP, EPCAM, SOD1 |
| ADGRE2, TSC1, MIR125A |
| MBTPS2, CDK4, VCAM1 |
| ANAPC1, BMPR1A, PGR-AS1 |
| RECQL4, MIR21, RMRP |
| PRKG1, AKT1, IL4 |
| RPS6KA1, TERT, SNORD15A |
| EDNRB, CCND1, BLM |
| MTAP, SDHA, KCNQ2 |
| TACR3, FLCN, IGF1R |
| CSNK2B, FANCC, PIK3R1 |
| TYR, FH, GRIN2A |
| AGER, HRAS, PARP1 |
| C2, FGFR2, TUBB3 |
| KCNJ6, ESR1, FANCA |
| MAD1L1, SDHB, KITLG |
| MC1R, MYC, TAP1 |
| CRBN, FGFR3, ATXN3 |
| CSNK2A2, AR, DCN |
| DDR1, LZTR1, HLA-B |
| ITGA1, HOTAIR, HLA-DRB1 |
| ITPR3, NRAS, IGFBP3 |
| KCNJ3, STAT3, NEU1 |
| RXRB, RUNX1, PLA2G6 |
| SFN, MEG3, POU5F1 |
| TYRP1, TGFBR2, ARID1A |
| HERC2, XRCC2, CFL1 |
| MAP3K11, MTOR, IL5 |
| PDE4B, UCA1, C4A |
| SLC34A2, MDM2, IL13 |
| BRD2, SMARCB1, SEMA3E |
| CDK10, PRKAR1A, HLA-DQA1 |
| DAXX, FGFR1, HLA-DQB1 |
| HLA-C, ERBB3, MSH5 |
| HLA-DPB1, MIR145, AIF1 |
| NRXN3, ERCC2, PCLO |
| OCA2, SRC, MIR877 |
| PKN2, NOTCH1, ITGB4 |
| PMEL, FBXW7, IFNA2 |
| ACSF3, RAD51, SLPI |
| COL11A2, EP300, CERNA3 |
| LTBP3, MIR155, SOD2-OT1 |
| PDCD6IP, TUG1, MMP2 |
| SEMA3C, MIR221, HMOX1 |
| SFRP4, MIR17, ABCB1 |
| STK3, MIR31, XIAP |
| VARS1, HIF1A, BCL2 |
| WNT7B, MIR143, BCL2L1 |
| ABCF1, MIR34A, CASP3 |
| CLIC1, IDH1, CDKN1B |
| FLOT1, ERBB4, SOD2 |
| IL1RL1, CHEK1, BIRC5 |
| MDC1, MAP2K1, CCNA2 |
| MRPS22, CASP8, SLC17A5 |
| NUDC, MAPK1, GPT |
| PCDH15, MIR214, RNASE3 |
| RIMS1, MIR30A, UGT1A1 |
| RING1, CDKN1A, RARB |
| RPL13, MIR195, NLRP3 |
| SEMA5A, MIR141, IL1A |
| SKIC2, MIR210, IGHE |
| SLC24A4, MIR146A, MEG8 |
| TRIM63, FANCD2, EGFR |
| VARS2, TGFB1, IFNG |
| DDX39B, CD274, IL1RN |
| DHDDS, BAX, JUN |
| HLA-E, GNAS, RARA |
| PACS1, PTGS2, TOP1 |
| BTG1, LINC-ROR, MTHFR |
| CAMTA1, PPARG, IRF1 |
| DHX16, PHOX2B, XDH |
| DIO2, MIR205, CXCL10 |
| EXOC2, WT1, RARS1 |
| HLA-DPA1, RAF1, CXCL9 |
| HLA-DRB5, MIR126, H19 |
| LTB, MIR27A, MALAT1 |
| MGAT5, NFE2L2, XIST |
| MICB, NTRK1, BDNF-AS |
| MYO3A, AURKA, MIR16-1 |
| NTM, MIR34C, LINC02605 |
| SLC45A2, CDKN2B, |
| TRIP11, MIR223, |
| TSC22D1, DHFR, |
| VAMP3, MIR20A, |
| ALG13, MIR106B, |
| APOM, EZH2, |
| ASIP, MIRLET7C, |
| B3GALT4, MIR146B, |
| BAG6, MIR29A, |
| DCLK2, ERCC4, |
| DGKB, MIR29C, |
| GAS8, MIR140, |
| GNG2, CREBBP, |
| GTF2B, MIR183, |
| HLA-DMA, FAS, |
| HLA-DMB, MIR139, |
| MICA, XRCC3, |
| PIGV, CXCR4, |
| SIPA1, PTPN11, |
| SLC24A5, MGMT, |
| SLC44A4, MIR10B, |
| SV2B, SMO, |
| ANKRD11, IL2, |
| HLA-DQA2, MIR18A, |
| IER3, MIR93, |
| LEMD2, MIR106A, |
| LSM2, MIR204, |
| MAB21L2, CD44, |
| PBX2, MIR22, |
| PPP1R10, MIR99A, |
| PPT2, MUC1, |
| RIN3, TERC, |
| RPS18, MIRLET7B, |
| TTC12, CYTOR, |
| WEE2, FASLG, |
| ZFP57, SMARCE1, |
| ATF6B, MIR150, |
| ATP6V0D2, PIK3CG, |
| CCHCR1, MIR181A1, |
| SEMA6D, EGFR-AS1, |
| ANXA9, XRCC1, |
| BNC2, MIR199A1, |
| DNAJC11, MIR142, |
| GTF2H4, MIR100, |
| HSD17B8, CDK2, |
| PIN4, FANCG, |
| RGL2, EGF, |
| SAA2, MIR149, |
| STK19, MIR373, |
| SYBU, KMT2D, |
| TAS2R38, CYP1A1, |
| TRIM39, MIR9-1, |
| HLA-DQB2, MKI67, |
| KLHL8, NFKB1, |
| NUFIP1, MIR23A, |
| SEPTIN8, NTRK2, |
| SORCS3, MIR98, |
| TENT4A, XPA, |
| UBAC2, GATA3, |
| VPS52, NKX2-1, |
| ZNF276, MIR19A, |
| ATP6V1G2, RASSF1, |
| BLTP1, KRT19, |
| CTSW, FANCL, |
| DPF3, SOX2, |
| DXO, MIR101-1, |
| EHBP1L1, YAP1, |
| GNL1, MIR122, |
| HMGN2, ERCC1, |
| LST1, PPP2R1A, |
| PRRC2A, ITGB1, |
| PTGR2, MIR193A, |
| SH3BGRL3, TNFRSF10B, |
| TCF25, LUCAT1, |
| ZNF100, PCNA, |
| ACOT2, DGCR5, |
| CPSF2, MT-CYB, |
| KCNK7, FANCF, |
| MUCL3, CDK6, |
| PPP1R18, IL10, |
| PRPF18, TP63, |
| PRSS58, PRKDC, |
| SGIP1, MIR185, |
| SNX32, MIR26B, |
| TCF19, MIR27B, |
| UBXN11, TYMS, |
| CFAP20, JAK2, |
| DBNDD1, FLT4, |
| GPSM3, CTLA4, |
| HUS1B, DLEC1, |
| NCKAP5, CYCS, |
| WDR46, IGF1, |
| ZNF778, MIRLET7E, |
| C6orf15, FHIT, |
| RNF217, MIR338, |
| STARD3NL, SOX9, |
| BEND7, MIR375, |
| EGFL8, DNMT1, |
| GPANK1, MIR125B1, |
| GPATCH3, PDCD1, |
| SPATA33, SMAD2, |
| TRIM10, MIR211, |
| VPS9D1, MIR331, |
| NHSL2, MIR29B2, |
| PCNX3, ZEB1, |
| PSORS1C1, MIR130A, |
| VWA7, PGR, |
| ZBTB12, MIR494, |
| DCAF4, SMAD3, |
| PRDM7, NME1, |
| TAS2R4, MIR15B, |
| TCERG1L, DNMT3B, |
| LY6G5B, CBS, |
| RPP21, VEGFC, |
| SAPCD1, NAT2, |
| OR9A4, FGF2, |
| PRR3, ING1, |
| SFTA2, MMP1, |
| SPATA2L, COMT, |
| PGPEP1L, ATRX, |
| TAS2R5, MIR423, |
| C6orf47, CFTR, |
| LY6G5C, MIR324, |
| MGAM2, CASC15, |
| MIR219A1, ADAR, |
| PRR23C, MIR124-1, |
| HCG22, AKT2, |
| HCG27, B2M, |
| SAA2-SAA4, FGF10, |
| LINC01619, DPYD, |
| LINC00243, MIR328, |
| LINC00304, CEACAM5, |
| HCG21, MIR30B, |
| HLA-F-AS1, HGF, |
| MSH5-SAPCD1, SETD2, |
| TH2LCRR, MIR132, |
| LINC00970, ABCG2, |
| HCG9, CASR, |
| MIR1236, MIRLET7A1, |
| TARP, MAP2K2, |
| TRG, MIR206, |
| LINC00486, PDGFRB, |
| MIR1976, GSK3B, |
| TPT1-AS1, OGG1, |
| LINC01425, CCND3, |
| LINC02569, SLC2A1, |
| SNORD68, TWIST1, |
| HLA-DQB1-AS1, LGALS3, |
| LINC01164, CXCL12, |
| LINC02274, FOXM1, |
| LINC02570, TP73, |
| BTG1-DT, CDKN3, |
| DDR1-DT, MIR425, |
| DHDDS-AS1, MIR193B, |
| LINC00330, E2F1, |
| LINC02219, MAPK3, |
| DDX39B-AS1, WEE1, |
| ENSG00000244255, CTSD, |
| LYPLAL1-DT, MIR485, |
| TSBP1-AS1, ICAM1, |
| ZNF593OS, MATR3, |
| LINC02823, ABL1, |
| LOC102723446, MIR483, |
| MIR3660, MIR361, |
| ZNF778-DT, ZEB2, |
| C6orf47-AS1, CDK1, |
| CAMTA1-AS3, HSP90AA1, |
| KCNQ5-DT, MIR486-1, |
| LINC01696, CYP2A6, |
| LINC01811, ALDH2, |
| LINC01937, CYP2E1, |
| SEMA5A-AS1, CSF2, |
| ENSG00000226698, PLAU, |
| ENSG00000229628, PTK2, |
| ENSG00000236266, SOS1, |
| ENSG00000261118, MIR144, |
| ENSG00000261172, HDAC1, |
| LINC01609, MIR197, |
| LINC01710, MCL1, |
| LINC02161, FAM3D-AS1, |
| LINC02964, CLPTM1L, |
| ENSG00000198211, MIR503, |
| ENSG00000205653, NSD1, |
| ENSG00000236452, DROSHA, |
| ENSG00000259828, PRF1, |
| ENSG00000261810, CASP9, |
| ENSG00000283321, MMP14, |
| ENSG00000201207, MIR370, |
| ENSG00000201555, MIR130B, |
| ENSG00000237773, CSF1R, |
| ENSG00000259006, PIK3CD, |
| ENSG00000259588, NQO1, |
| ENSG00000268218, MIR33A, |
| LOC124909439, BMP4, |
| FKSG29, FN1, |
| LINC02849, CD4, |
| LOC105375170, MIR128-1, |
| ENSG00000286452, CSF3, |
| ENSG00000289375, TNFSF10, |
| HLA-DQA1-AS1, SNAI1, |
| HSALNG0020226-409, RAC1, |
| HSALNG0149394, GSTM3, |
| HSALNG0149400, WRAP53, |
| L13304-005, TNFRSF1A, |
| L13715-004, CCNB1, |
| LOC101927556, TIMP1, |
| LOC107986954, MMP7, |
| LOC124902693, ARAF, |
| NONHSAG011893.2, TOP2A, |
| lnc-BTG1-3, MIR135B, |
| lnc-DDR1-4, EPHB4, |
| lnc-FOXL2NB-8, SP1, |
| lnc-LSM2-1, CAV1, |
| lnc-MC1R-1, ADH1C, |
| lnc-NCR3-2, MIR138-1, |
| CM034953-072, HMGA2, |
| CM034956-102, ALB, |
| CM034957-065, BMI1, |
| ENSG00000267048, TBX1, |
| HSALNG0004215, DELEC1, |
| HSALNG0049329, TYMP, |
| HSALNG0056376, SOCS1, |
| HSALNG0058876-012, PLK1, |
| HSALNG0084920, VIM, |
| HSALNG0108511, TGFA, |
| HSALNG0109497, APEX1, |
| HSALNG0142211, FGF3, |
| HSALNG0149401, MIR186, |
| KR024017, HSPB1, |
| LOC105378797, MIR675, |
| LOC124903404, MIR532, |
| LOC124903759, FOXP3, |
| MN298114-173, LMNA, |
| NONHSAG020376.2, MIR137, |
| NONHSAG043593.2, LEF1, |
| NONHSAG046469.2, NOTCH3, |
| RF00017-5450, MPO, |
| RF00017-5465, SF3B2, |
| RF00017-5468, HSPA4, |
| RF00017-5469, TIMP2, |
| RF00066-145, PDGFB, |
| hsa-miR-1273e-047, ADH1B, |
| hsa-miR-5095-422, CCL2, |
| lnc-CFL1-2, CSF1, |
| lnc-GAS8-6, EPHA2, |
| lnc-KCNK7-1, HLA-A, |
| lnc-MAB21L2-6, MIR376A1, |
| lnc-PRDM7-3, CYP2D6, |
| lnc-RPS6KA1-1, PROM1, |
| lnc-ZFYVE1-6, ENO2, |
| piR-43106-125, IFNA1, |
| piR-50241-003, TYK2, |
| HQ292177, HMGB1, |
| HSALNG0001909-001, ELANE, |
| HSALNG0001909-002, CTCF, |
| HSALNG0047287, PTK2B, |
| HSALNG0047288, PLG, |
| HSALNG0049465, CD40LG, |
| HSALNG0049494, SNAI2, |
| HSALNG0056379, NFKB2, |
| HSALNG0067748, EDN1, |
| HSALNG0102314, CYP3A4, |
| HSALNG0103263, BSG, |
| HSALNG0105719, PLAUR, |
| LOC105374453, KRT5, |
| LOC124906290, MIR330, |
| NONHSAG045689.2-003, MUC5AC, |
| NONHSAG048989.2, PAX8, |
| RF00017-024, STAT5B, |
| lnc-BRD2-2, BAK1, |
| lnc-BRD2-3, FGF4, |
| lnc-BRD2-4, CD8A, |
| piR-31199-104, PIK3CB, |
| piR-33804-058, MIR20B, |
| piR-34093-008, CDX2, |
| piR-35002-055, TNFRSF10A, |
| piR-43583-467, PDCD1LG2, |
| piR-44759, NOD2, |
| piR-45471-516, TGFB2, |
| piR-54420, EBAG9, |
| piR-55361-380, CRKL, |
| piR-55654-435, MTR, |
| CM034963-089, BDNF, |
| HSALNG0001895, CD36, |
| HSALNG0049430, FSCN1, |
| HSALNG0087239, PTHLH, |
| HSALNG0096774, LDHA, |
| HSALNG0096776, CTTN, |
| LOC105375264, CXCR2, |
| LOC105378178, HDAC4, |
| RF00017-2203, DSP, |
| lnc-MICA-7, CCL5, |
| lnc-VPS9D1-3, PDPN, |
| LOC105373696, CALCA, |
| LOC124901594, AREG, |
| LOC124905383, FADD, |
| NONHSAG055113.2, ALDH1A1, |
| MRGPRX2, TBX3, |
| RELA, RPSA, |
| MAPK8, LGALS1, |
| NOS2, FOXE1, |
| STIM1, HEIH, |
| BIRC2, MCM4, |
| GJB1, EZR, |
| BCL2L11, HDAC9, |
| BIRC3, THBS1, |
| BGLAP, INS, |
| TRP-AGG2-5, DSG3, |
| MEN1, EIF4E, |
| NR1H2, RPS6KB1, |
| KLK7, FGF8, |
| KLK5, MMP11, |
| PRKCA, SOX4, |
| G6PD, RREB1, |
| ANPEP, EPHX1, |
| CREB1, IDO1, |
| RXRA, CDH2, |
| FAP, CD28, |
| P2RX4, MIR133A1, |
| KRT10, CTSB, |
| P2RY2, EGR1, |
| FLG, CYP1A2, |
| SHBG, WNT5A, |
| DPP9, BMP2, |
| IL18BP, NRP1, |
| RXRG, CCR7, |
| IL36G, TIMP3, |
| IVL, SHH, |
| PI3, PML, |
| MIR16-2, HSP90AB1, |
| VTRNA1-2, ACTB, |
| TRV-CAC1-2, ECM1, |
| SST, |
| CP, |
| SIRT1, |
| ITGA6, |
| LZTS1, |
| GAPDH, |
| KRT18, |
| CHGA, |
| YY1, |
| MIR29B1, |
| BECN1, |
| ADA, |
| KLF4, |
| MIR512-1, |
| CA9, |
| JAG1, |
| CCN2, |
| PRKD1, |
| IRAK4, |
| IL2RA, |
| FBN1, |
| NCAM1, |
| CD80, |
| SYP, |
| ANXA1, |
| VCAN, |
| SF3B3, |
| MAGEA1, |
| CD24, |
| NAT1, |
| AFP, |
| REV3L, |
| RD4, |
| KRT14, |
| KLRK1, |
| PAH, |
| MIR154, |
| GRP, |
| TGM2, |
| MIR135A1, |
| CXCL1, |
| FUS, |
| ITGA5, |
| JUP, |
| PTH, |
| SQSTM1, |
| ANXA5, |
| KCNH2, |
| ACE, |
| MMP13, |
| HPSE, |
| ACTC1, |
| APOB, |
| IFNB1, |
| ANO3, |
| POSTN, |
| CD46, |
| SERPINA1, |
| MIR379, |
| XRCC6, |
| MSLN, |
| TFRC, |
| EIF4EBP1, |
| SULT1A1, |
| CLDN7, |
| HSPD1, |
| GZMB, |
| FGF1, |
| YWHAE, |
| CDH13, |
| F3, |
| LOX, |
| PTPRC, |
| ANXA2, |
| LINC01554, |
| SOX10, |
| IL15, |
| LTA, |
| LYN, |
| IKBKB, |
| MIR574, |
| FEN1, |
| PTGS1, |
| CASP1, |
| SFTPA2, |
| TKT, |
| RPS27A, |
| ASAH1, |
| CAT, |
| MAF, |
| CD86, |
| SAMHD1, |
| CDR1-AS, |
| ATP7B, |
| CCNA1, |
| HNF4A, |
| ITGA3, |
| HLA-G, |
| CD27, |
| FGF9, |
| CD34, |
| GNAL, |
| MYRF, |
| MECP2, |
| MIR382, |
| HSPG2, |
| NANOG, |
| HSPA8, |
| SERPINB2, |
| LACTB, |
| MIR615, |
| SPON2, |
| EPIST, |
| CYP2C19, |
| ATF3, |
| CD9, |
| GIPC1, |
| APOE, |
| TUBB4A, |
| TPP1, |
| VTN, |
| CXCR3, |
| PPARD, |
| ZBTB7A, |
| MIR744, |
| TF, |
| IL2RB, |
| TGFB3, |
| CD79A, |
| TNC, |
| POLB, |
| SERPINB3, |
| IGSF9B, |
| MIR362, |
| GLI3, |
| KLK6, |
| PECAM1, |
| MIR506 |
| MSN |
| TACSTD2 |
| MAGEA4 |
| ARID1B |
| PLAT |
| MMP12 |
| EIF2AK2 |
| TAF1 |
| MIR493 |
| SELE |
| CTSL |
| IL1R1 |
| KNG1 |
| SLC16A1 |
| CCR5 |
| TRA-TGC7-1 |
| MIR654 |
| IKBKG |
| CRNN |
| EIF2AK3 |
| TUBB |
| CD47 |
| POMC |
| CLDN3 |
| LIF |
| ACVR1 |
| NRP2 |
| CXCL5 |
| PIK3C3 |
| PPP2CA |
| HES1 |
| KAT6A |
| RAG2 |
| PSMB8 |
| ALPP |
| LAG3 |
| MBL2 |
| FURIN |
| SLC2A3 |
| NUTM1 |
| LRP12 |
| H3-3B |
| LRP1 |
| CXADR |
| VRK1 |
| ADH5 |
| SERPING1 |
| MIR490 |
| GNE |
| WNT10B |
| ADM |
| ARG1 |
| HAX1 |
| TRAF6 |
| AICDA |
| TREX1 |
| PARK7 |
| GATA6 |
| LAMP1 |
| HK1 |
| SIX1 |
| TAC1 |
| MIR33B |
| ZAP70 |
| THAP1 |
| CD59 |
| MIR363 |
| SPECC1L |
| CCL3 |
| DRD2 |
| TTR |
| HNRNPA1 |
| WNT3A |
| CYP2C9 |
| MMP10 |
| KMT2B |
| SUMF1 |
| MAPT |
| HPRT1 |
| ACP1 |
| H3-4 |
| MYOD1 |
| GSR |
| DGCR8 |
| PTPN13 |
| PRKRA |
| LINC01194 |
| HIRA |
| GLI2 |
| ANO1 |
| FLNB |
| ACE2 |
| ALDH9A1 |
| UGT1A7 |
| SERPINA3 |
| PPARGC1A |
| CTSK |
| ITGAM |
| TUSC3 |
| PSAP |
| CHD7 |
| CDH11 |
| RNASEH2A |
| NOG |
| FLNA |
| FOXC2 |
| SELENON |
| CD58 |
| CSTA |
| GLA |
| ELN |
| CANX |
| PSMB9 |
| RNASEH2B |
| LGALS9 |
| JMJD1C |
| NOTCH2NLC |
| NFIX |
| DDX53 |
| MIR885 |
| UFD1 |
| VCP |
| GP1BB |
| CD1A |
| IFIH1 |
| EIF4G1 |
| ITGB2 |
| TPI1 |
| SNCA |
| MYOM2 |
| CKB |
| KDM4C |
| IL22 |
| MIR545 |
| APOA1 |
| RPL10 |
| RILPL1 |
| CD68 |
| MYH14 |
| LIG3 |
| UCHL1 |
| TUBA1B |
| P2RX7 |
| PABPN1 |
| CCL22 |
| BCL2L12 |
| HMBS |
| CR2 |
| TFEB |
| ARVCF |
| LTO1 |
| AQP1 |
| RIGI |
| GFAP |
| HYAL1 |
| MUC5B |
| GLB1 |
| COL2A1 |
| CALB2 |
| LTF |
| CYP2C8 |
| COL17A1 |
| SEC24C |
| LOXL2 |
| LAMA2 |
| KRT1 |
| VCL |
| CACNA1S |
| KRT13 |
| IREB2 |
| COX5A |
| UBE3A |
| PC |
| FMR1 |
| PAX6 |
| ANXA11 |
| CD63 |
| CIZ1 |
| S100B |
| PAX7 |
| GDNF |
| HSD17B4 |
| CD69 |
| MAGT1 |
| LPL |
| RNASEH2C |
| TBCE |
| RNU6-1 |
| SELP |
| GGT1 |
| PRAME |
| CHD4 |
| GRM1 |
| TOR1A |
| SELL |
| MAL |
| MID1 |
| EYA1 |
| NTF3 |
| TRPV1 |
| CCL11 |
| S100A1 |
| NPY |
| TANGO2 |
| LBR |
| HIC2 |
| MAP2K7 |
| SIRT2 |
| DES |
| HBB |
| SMPD1 |
| XPO5 |
| APP |
| CTNNBIP1 |
| HEPACAM |
| FGF14 |
| CACNA1G |
| HCCAT5 |
| TMC8 |
| PRNP |
| PRKCG |
| TBP |
| PAX9 |
| SATB2 |
| C1S |
| MIR874 |
| TBX5 |
| SFTPA1 |
| GBA1 |
| EMP1 |
| GALC |
| CXCL2 |
| NPC1 |
| F12 |
| CAV2 |
| IRF6 |
| HADHA |
| FXN |
| PPP1CA |
| COMMD3-BMI1 |
| C3 |
| DKK4 |
| AKR1A1 |
| FUCA1 |
| CNR1 |
| WTAP |
| TACR1 |
| GBX2 |
| AMBP |
| CD99 |
| CACNA1A |
| TSLP |
| SPECC1L-ADORA2A |
| MFN2 |
| ELP1 |
| SCGB1A1 |
| ACHE |
| MIB1 |
| MYOG |
| SLC34A1 |
| SFTPC |
| KRT4 |
| LTBP1 |
| SLC11A1 |
| OFD1 |
| MIR766 |
| KCNA3 |
| ARSA |
| KRT15 |
| SFTPB |
| ITGAL |
| TMC6 |
| MLC1 |
| SETX |
| VPS13B |
| AOC3 |
| SCARF2 |
| DLG1 |
| TCOF1 |
| IL9 |
| IGKC |
| RBM8A |
| ACKR3 |
| PTPN22 |
| PGD |
| GRK2 |
| PSEN1 |
| PSMA3 |
| IL18R1 |
| APTX |
| PRTN3 |
| MSX1 |
| RNU4ATAC |
| AIRE |
| DMAP1 |
| NOD1 |
| CD2 |
| HTT |
| TH |
| SFTPD |
| ATP1A3 |
| KCNH1 |
| TNFSF13 |
| CD1D |
| ATXN2 |
| ARG2 |
| OXT |
| FTH1 |
| HTR2A |
| TRAT1 |
| RAI1 |
| SMN1 |
| MC2R |
| FAAH |
| CASK |
| BDKRB2 |
| PDLIM7 |
| C5AR1 |
| GATA5 |
| MB |
| GNAI3 |
| TARDBP |
| ZIC3 |
| TUBA1A |
| OPRM1 |
| EXOSC3 |
| ABCB7 |
| CRYAB |
| DEFB4A |
| SPTBN2 |
| DST |
| CYP2A13 |
| SPG7 |
| CFHR5 |
| BAGE |
| H6PD |
| HEXB |
| WNT10A |
| F13A1 |
| GJB5 |
| PIK3C2A |
| IGBP1 |
| KLKB1 |
| EXOSC6 |
| CKMT1B |
| POLG |
| ERF |
| GCM2 |
| PPP2R2B |
| COL9A3 |
| ITPR1 |
| ASPA |
| CCL17 |
| SNAP29 |
| DHCR7 |
| PABPC1 |
| PLOD2 |
| TCIRG1 |
| DHCR24 |
| ARHGAP29 |
| PIGA |
| MYH6 |
| SLC11A2 |
| WFS1 |
| GRHL3 |
| CHAF1A |
| SCN2A |
| IL12RB2 |
| GALNS |
| ALPL |
| PURA |
| SERPINC1 |
| VPS13A |
| RTN4R |
| GOLGA4 |
| FGFBP1 |
| LOXL4 |
| PLP1 |
| ADH7 |
| SLC52A3 |
| ADGRG1 |
| RRM2B |
| OLIG2 |
| CNTNAP2 |
| PHIP |
| TMCO1 |
| AFG3L2 |
| SLC22A5 |
| CARMN |
| RAB5A |
| PANK2 |
| ATXN10 |
| NEUROG1 |
| NAGLU |
| FTL |
| MGP |
| TCF20 |
| TRIM8 |
| EXOSC9 |
| GNPTAB |
| COQ8A |
| DEAF1 |
| TRIP10 |
| BAZ1B |
| VAPB |
| SCN4A |
| SOX3 |
| PLEC |
| HEXA |
| CSTB |
| DGCR2 |
| KANSL1 |
| EVC2 |
| FERMT3 |
| POLR3A |
| ABCD1 |
| TEAD4 |
| ATXN7 |
| TRIM32 |
| CR1 |
| AGTPBP1 |
| CACNA1D |
| GRIA3 |
| C19orf12 |
| HRH1 |
| LHPP |
| TECR |
| AVP |
| LGI1 |
| SGCE |
| AARS1 |
| MBP |
| POLR3B |
| DHODH |
| KCNMA1 |
| MMS19 |
| FCHO1 |
| GSTA2 |
| DAB1 |
| XPNPEP2 |
| CNTF |
| RPN2 |
| CELF1 |
| MCOLN1 |
| GJC2 |
| EXTL3 |
| DPM1 |
| GRID2 |
| CKM |
| UBR4 |
| PKP1 |
| CACNA1C |
| CHN1 |
| TRPV3 |
| GCH1 |
| STX16 |
| SMN2 |
| SH2D3A |
| DEFA5 |
| PEX19 |
| MAG |
| TKTL2 |
| PABPC4 |
| ATXN8OS |
| RBFOX3 |
| EMC1 |
| CTSA |
| EIF2B2 |
| BTD |
| COASY |
| RPL7A |
| EIF2B1 |
| NEFL |
| COL9A1 |
| HHAT |
| TREM1 |
| CKMT2 |
| IL1RAPL2 |
| FGB |
| SLC25A6 |
| GBE1 |
| STXBP1 |
| DLD |
| SPAST |
| ALDH1B1 |
| PRRT2 |
| IDS |
| AFF2 |
| ETFA |
| GJB6 |
| ADCY6 |
| STRA6 |
| POLR2B |
| HTR1A |
| PI4KA |
| PNKD |
| GNPAT |
| FLII |
| MASP1 |
| SLC1A3 |
| ZMPSTE24 |
| EFTUD2 |
| HAAO |
| CNTNAP1 |
| XK |
| HHEX |
| MASP2 |
| DARS1 |
| AHDC1 |
| DLX5 |
| SARDH |
| ALMS1 |
| HGSNAT |
| SERPINB1 |
| FABP12 |
| TLX1NB |
| TRPA1 |
| MIR802 |
| MAP3K9 |
| SNRPB |
| ATN1 |
| PVALB |
| GCDH |
| SRPX2 |
| TRIM17 |
| FIG4 |
| SNRPN |
| SETD1B |
| PEX2 |
| ACO1 |
| SKIC3 |
| PEX14 |
| PCCB |
| GON4L |
| TSEN15 |
| ARX |
| EDAR |
| SCN1A |
| RAB11A |
| SH2D3C |
| ATP1A2 |
| FA2H |
| PEX5 |
| PCCA |
| DARS2 |
| OGA |
| ADSL |
| ATP1B1 |
| PRPF40A |
| CLMN |
| IL31 |
| PPFIA1 |
| TBX4 |
| POLR3K |
| GAD1 |
| SEPSECS |
| ATXN1 |
| PEX10 |
| SLC25A46 |
| HCRT |
| ACOX1 |
| LY6D |
| TRIM2 |
| SGSH |
| TRPV2 |
| GTPBP1 |
| KCNA4 |
| EXOSC8 |
| ANTXR2 |
| TBX6 |
| HARS1 |
| UGT8 |
| C1R |
| TNFAIP2 |
| GFER |
| PITX1 |
| HTR1B |
| CNP |
| BBOX1 |
| ZNF699 |
| DNAAF5 |
| PEX1 |
| NPBWR1 |
| OR8J1 |
| SLC1A2 |
| POM121 |
| GHSR |
| L2HGDH |
| TSEN2 |
| INVS |
| GSTT2 |
| PAFAH1B1 |
| NPC2 |
| IDUA |
| TRIM36 |
| PFDN4 |
| RAI14 |
| TSEN34 |
| RCAN1 |
| FOXJ1 |
| LMLN |
| KCNA1 |
| ETFDH |
| SLC16A2 |
| PRPH |
| PDZK1IP1 |
| ACADM |
| MYMK |
| ADAMTSL2 |
| LBP |
| MAN2B1 |
| PSMD2 |
| NDUFAF2 |
| EXOSC10 |
| EDA |
| SLC20A2 |
| ETFB |
| NDUFS4 |
| SSR1 |
| ARHGEF17 |
| ACADVL |
| MARCHF6 |
| MPI |
| AARS2 |
| APRT |
| SLC52A2 |
| RAPGEF2 |
| ADH6 |
| TBC1D24 |
| NOTCH2NLA |
| MYH2 |
| AZU1 |
| TSEN54 |
| CACNA1B |
| LINC00328 |
| PDE4A |
| GLE1 |
| DLX3 |
| CFAP57 |
| C1QC |
| HADH |
| PQBP1 |
| NUTM2B-AS1 |
| CECR2 |
| ANKK1 |
| SAA4 |
| HYCC1 |
| GTF3C1 |
| CYP51A1 |
| ADK |
| DYNLL1 |
| CPM |
| SNAP25 |
| C4B |
| BCKDHA |
| PEX7 |
| SLC1A1 |
| MYF5 |
| RAB6A |
| QDPR |
| GM2A |
| TRIM9 |
| CACNA1H |
| PEX6 |
| COLEC11 |
| CPSF4 |
| KCND3 |
| DDC |
| ACTRT1 |
| MID2 |
| EXOSC4 |
| PEX3 |
| ACR |
| SPG11 |
| BARX1 |
| JPH3 |
| PEX16 |
| EARS2 |
| PPT1 |
| CHRD |
| KCNA10 |
| SLC19A3 |
| KCNA2 |
| CFHR2 |
| ALG3 |
| KIFBP |
| KCNJ10 |
| SYT16 |
| PEX26 |
| GAD2 |
| SLC34A3 |
| PEX11B |
| SYNM |
| MCCC1 |
| EXOSC5 |
| EXOSC7 |
| PANK1 |
| ENSG00000276861 |
| SAMD12 |
| ZSCAN1 |
| D2HGDH |
| SCN8A |
| MTM1 |
| GALE |
| KCNAB2 |
| NAGA |
| TMEM106B |
| PEX12 |
| MDFIC |
| PEX13 |
| ZXDB |
| RBM27 |
| EBP |
| CHAT |
| SUOX |
| CAV3 |
| EXOSC2 |
| DHPS |
| KIF21A |
| MOCS1 |
| ZSCAN21 |
| COL25A1 |
| CHRNA6 |
| ATP13A2 |
| CHRNB3 |
| KCNQ3 |
| HCCS |
| RBM7 |
| CFHR4 |
| TRIM67 |
| PLXND1 |
| PRY2 |
| BCKDHB |
| EXOSC1 |
| BHLHA9 |
| KCND2 |
| PCDHA13 |
| KRT27 |
| QRICH1 |
| CLN8 |
| TRIM54 |
| TMEM126A |
| PIGK |
| HMGCL |
| TTBK2 |
| DBT |
| PIGN |
| HLCS |
| IGKV1-33 |
| CLN6 |
| CYLD-AS1 |
| KCNC3 |
| KDF1 |
| KCNB1 |
| PMM2 |
| TMEM178A |
| EDARADD |
| TSPEAR |
| KLHL15 |
| TXNL4A |
| SLC9A3 |
| IQSEC2 |
| CACNB4 |
| EDA2R |
| DCAF17 |
| DNAI2 |
| CACNA1E |
| SEMA6C |
| GABRG2 |
| MIR676 |
| TPH1 |
| IGKV1D-33 |
| TMEM63A |
| MTMR11 |
| PRY |
| WDR45 |
| TMEM260 |
| KCNC4 |
| MOCS2 |
| GAMT |
| LOXL3 |
| KATNIP |
| CLN3 |
| KCNK18 |
| CACNA1I |
| P2RX3 |
| OPA3 |
| CLN5 |
| GTPBP2 |
| TIMM8A |
| SCN1B |
| HIKESHI |
| TRAF3IP1 |
| NALCN |
| KCNAB1 |
| ZFAND4 |
| STAC3 |
| TNNC2 |
| SLC1A6 |
| CACNA1F |
| POM121L12 |
| KIR2DS1 |
| KCNC1 |
| KIR2DS5 |
| PCDH19 |
| KIR3DS1 |

**Table S4**

| **Category** | **Term** | **Count** | **%** | **p-value** | **Genes** | **List Total** | **Pop Hits** | **Pop Total** | **Fold Enrichment** | **Bonferroni** | **Benjamini** | **FDR** |
| --- | --- | --- | --- | --- | --- | --- | --- | --- | --- | --- | --- | --- |
| KEGG_PATHWAY | hsa05321:Inflammatory bowel disease | 17 | 14,91228 | 7.87E-19 | JUN, STAT1, IL13, IL18, TNF, IL4, IL1A, IL6, IL5, IFNG, IL1B, TLR4, HLA-DQA1, HLA-DRB1, HLA-DQB1, IL17A, TLR2 | 88 | 65 | 8840 | 26,27273 | 1.86E-16 | 1.86E-16 | 1.16E-16 |
| KEGG_PATHWAY | hsa05323:Rheumatoid arthritis | 17 | 14,91228 | 4.40E-16 | JUN, FLT1, CXCL8, MMP3, IL18, TNF, VEGFA, IL1A, IL6, IFNG, IL1B, TLR4, HLA-DQA1, HLA-DRB1, HLA-DQB1, IL17A, TLR2 | 88 | 94 | 8840 | 18,16731 | 1.05E-13 | 5.19E-14 | 3.26E-14 |
| KEGG_PATHWAY | hsa05164:Influenza A | 20 | 17,54386 | 2.50E-15 | IL33, CXCL8, STAT1, IFNA2, IL18, PIK3R1, TNF, IL1A, CXCL10, IL6, IFNG, IL1B, CASP3, NLRP3, TLR4, MYD88, HLA-DQA1, HLA-DRB1, TLR3, HLA-DQB1 | 88 | 172 | 8840 | 11,68076 | 6.03E-13 | 1.97E-13 | 1.23E-13 |
| KEGG_PATHWAY | hsa05200:Pathways in cancer | 29 | 25,4386 | 6.18E-14 | CDKN1B, CXCL8, GSTP1, IFNA2, XIAP, GSTT1, PIK3R1, EGFR, IGF1R, CASP3, HMOX1, JUN, GSTM1, STAT1, MMP2, IL13, MMP9, VEGFA, IL4, CCNA2, KITLG, IL6, IL5, IFNG, BCL2, RARA, BIRC5, RARB, BCL2L1 | 88 | 533 | 8840 | 5,465632 | 1.46E-11 | 3.64E-12 | 2.29E-12 |
| KEGG_PATHWAY | hsa04657:IL-17 signaling pathway | 15 | 13,15789 | 2.92E-13 | JUN, CXCL8, IL13, MMP3, TNF, MMP9, IL4, CXCL10, IL6, IL5, IFNG, IL1B, CASP3, S100A8, IL17A | 88 | 95 | 8840 | 15,86124 | 6.88E-11 | 1.38E-11 | 8.63E-12 |
| KEGG_PATHWAY | hsa04620:Toll-like receptor signaling pathway | 15 | 13,15789 | 2.05E-12 | JUN, CXCL9, CXCL8, STAT1, IFNA2, PIK3R1, TNF, CXCL10, IL6, IL1B, SPP1, TLR4, MYD88, TLR3, TLR2 | 88 | 109 | 8840 | 13,82402 | 4.84E-10 | 6.95E-11 | 4.36E-11 |
| KEGG_PATHWAY | hsa05417:Lipid and atherosclerosis | 19 | 16,66667 | 2.06E-12 | JUN, VCAM1, CXCL8, IFNA2, MMP3, IL18, PIK3R1, SOD2, TNF, MMP9, IL6, IL1B, CASP3, BCL2, NLRP3, TLR4, MYD88, BCL2L1, TLR2 | 88 | 216 | 8840 | 8,836279 | 4.87E-10 | 6.95E-11 | 4.36E-11 |
| KEGG_PATHWAY | hsa05418:Fluid shear stress and atherosclerosis | 16 | 14,03509 | 5.13E-12 | JUN, GSTM1, VCAM1, GSTP1, MMP2, GSTT1, PIK3R1, TNF, MMP9, VEGFA, IL1A, IFNG, IL1B, KDR, BCL2, HMOX1 | 88 | 141 | 8840 | 11,3991 | 1.21E-09 | 1.51E-10 | 9.48E-11 |
| KEGG_PATHWAY | hsa05140:Leishmaniasis | 13 | 11,40351 | 8.27E-12 | JUN, STAT1, TNF, IL4, IL1A, IFNG, IL1B, TLR4, MYD88, HLA-DQA1, HLA-DRB1, HLA-DQB1, TLR2 | 88 | 77 | 8840 | 16,95986 | 1.95E-09 | 2.00E-10 | 1.25E-10 |
| KEGG_PATHWAY | hsa05169:Epstein-Barr virus infection | 18 | 15,78947 | 8.46E-12 | JUN, CDKN1B, STAT1, IFNA2, HLA-B, TAP1, PIK3R1, TNF, CCNA2, CXCL10, IL6, CASP3, BCL2, MYD88, HLA-DQA1, HLA-DRB1, HLA-DQB1, TLR2 | 88 | 203 | 8840 | 8,9073 | 2.00E-09 | 2.00E-10 | 1.25E-10 |
| KEGG_PATHWAY | hsa05133:Pertussis | 13 | 11,40351 | 9.70E-12 | JUN, CXCL8, TNF, IL1A, C4A, IL6, IL1B, CASP3, IRF1, CFL1, NLRP3, TLR4, MYD88 | 88 | 78 | 8840 | 16,74242 | 2.29E-09 | 2.08E-10 | 1.30E-10 |
| KEGG_PATHWAY | hsa04933:AGE-RAGE signaling pathway in diabetic complications | 14 | 12,2807 | 1.31E-11 | JUN, CDKN1B, VCAM1, CXCL8, STAT1, MMP2, PIK3R1, TNF, VEGFA, IL1A, IL6, IL1B, CASP3, BCL2 | 88 | 101 | 8840 | 13,92439 | 3.09E-09 | 2.57E-10 | 1.61E-10 |
| KEGG_PATHWAY | hsa05161:Hepatitis B | 16 | 14,03509 | 4.23E-11 | JUN, CXCL8, STAT1, IFNA2, PIK3R1, TNF, MMP9, CCNA2, IL6, CASP3, BCL2, BIRC5, TLR4, MYD88, TLR3, TLR2 | 88 | 163 | 8840 | 9,860569 | 9.99E-09 | 7.69E-10 | 4.82E-10 |
| KEGG_PATHWAY | hsa05152:Tuberculosis | 16 | 14,03509 | 1.75E-10 | STAT1, IFNA2, IL18, TNF, IL1A, IL6, IFNG, IL1B, CASP3, BCL2, TLR4, MYD88, HLA-DQA1, HLA-DRB1, HLA-DQB1, TLR2 | 88 | 180 | 8840 | 8,929293 | 4.14E-08 | 2.95E-09 | 1.85E-09 |
| KEGG_PATHWAY | hsa05145:Toxoplasmosis | 13 | 11,40351 | 6.78E-10 | STAT1, XIAP, TNF, IFNG, CASP3, BCL2, TLR4, MYD88, HLA-DQA1, HLA-DRB1, HLA-DQB1, BCL2L1, TLR2 | 88 | 111 | 8840 | 11,76495 | 1.60E-07 | 1.07E-08 | 6.69E-09 |
| KEGG_PATHWAY | hsa05162:Measles | 14 | 12,2807 | 7.73E-10 | JUN, CDKN1B, STAT1, IFNA2, PIK3R1, IL1A, IL6, IL1B, CASP3, BCL2, TLR4, MYD88, BCL2L1, TLR2 | 88 | 139 | 8840 | 10,11772 | 1.83E-07 | 1.14E-08 | 7.15E-09 |
| KEGG_PATHWAY | hsa05171:Coronavirus disease - COVID-19 | 17 | 14,91228 | 9.96E-10 | JUN, CXCL8, STAT1, IFNA2, MMP3, PIK3R1, TNF, EGFR, CXCL10, C4A, IL6, IL1B, NLRP3, TLR4, MYD88, TLR3, TLR2 | 88 | 238 | 8840 | 7,175325 | 2.35E-07 | 1.34E-08 | 8.42E-09 |
| KEGG_PATHWAY | hsa05144:Malaria | 10 | 8,77193 | 1.02E-09 | IL6, VCAM1, CXCL8, IFNG, IL1B, IL18, TNF, TLR4, MYD88, TLR2 | 88 | 50 | 8840 | 20,09091 | 2.42E-07 | 1.34E-08 | 8.42E-09 |
| KEGG_PATHWAY | hsa05332:Graft-versus-host disease | 9 | 7,894737 | 8.43E-09 | IL1A, IL6, IFNG, IL1B, HLA-B, TNF, HLA-DRB1, HLA-DQA1, HLA-DQB1 | 88 | 44 | 8840 | 20,54752 | 1.99E-06 | 1.05E-07 | 6.56E-08 |
| KEGG_PATHWAY | hsa05310:Asthma | 8 | 7,017544 | 1.52E-08 | IL4, IL5, IL13, RNASE3, TNF, HLA-DRB1, HLA-DQA1, HLA-DQB1 | 88 | 31 | 8840 | 25,92375 | 3.59E-06 | 1.79E-07 | 1.13E-07 |
| KEGG_PATHWAY | hsa04668:TNF signaling pathway | 12 | 10,52632 | 1.95E-08 | CXCL10, IL6, JUN, VCAM1, IL1B, CASP3, IRF1, MMP3, XIAP, PIK3R1, TNF, MMP9 | 88 | 119 | 8840 | 10,12987 | 4.61E-06 | 2.19E-07 | 1.38E-07 |
| KEGG_PATHWAY | hsa04621:NOD-like receptor signaling pathway | 14 | 12,2807 | 3.31E-08 | JUN, CXCL8, STAT1, IFNA2, IL18, XIAP, TNF, IL6, IL1B, BCL2, NLRP3, TLR4, MYD88, BCL2L1 | 88 | 189 | 8840 | 7,441077 | 7.81E-06 | 3.55E-07 | 2.23E-07 |
| KEGG_PATHWAY | hsa04217:Necroptosis | 13 | 11,40351 | 4.19E-08 | IL33, PARP1, STAT1, IFNA2, XIAP, TNF, IL1A, IFNG, IL1B, BCL2, NLRP3, TLR4, TLR3 | 88 | 159 | 8840 | 8,213265 | 9.88E-06 | 4.30E-07 | 2.69E-07 |
| KEGG_PATHWAY | hsa05134:Legionellosis | 9 | 7,894737 | 6.14E-08 | IL6, CXCL8, IL1B, CASP3, IL18, TNF, TLR4, MYD88, TLR2 | 88 | 56 | 8840 | 16,14448 | 1.45E-05 | 6.03E-07 | 3.78E-07 |
| KEGG_PATHWAY | hsa05330:Allograft rejection | 8 | 7,017544 | 6.90E-08 | IL4, IL5, IFNG, HLA-B, TNF, HLA-DRB1, HLA-DQA1, HLA-DQB1 | 88 | 38 | 8840 | 21,14833 | 1.63E-05 | 6.52E-07 | 4.09E-07 |
| KEGG_PATHWAY | hsa04659:Th17 cell differentiation | 11 | 9,649123 | 8.88E-08 | IL4, IL6, JUN, IFNG, STAT1, IL1B, RARA, HLA-DRB1, HLA-DQA1, IL17A, HLA-DQB1 | 88 | 108 | 8840 | 10,23148 | 2.09E-05 | 8.06E-07 | 5.05E-07 |
| KEGG_PATHWAY | hsa04066:HIF-1 signaling pathway | 11 | 9,649123 | 9.70E-08 | IL6, FLT1, CDKN1B, IFNG, BCL2, HMOX1, PIK3R1, TLR4, EGFR, IGF1R, VEGFA | 88 | 109 | 8840 | 10,13761 | 2.29E-05 | 8.47E-07 | 5.31E-07 |
| KEGG_PATHWAY | hsa04940:Type I diabetes mellitus | 8 | 7,017544 | 1.69E-07 | IL1A, IFNG, IL1B, HLA-B, TNF, HLA-DRB1, HLA-DQA1, HLA-DQB1 | 88 | 43 | 8840 | 18,68922 | 4.00E-05 | 1.39E-06 | 8.72E-07 |
| KEGG_PATHWAY | hsa04060:Cytokine-cytokine receptor interaction | 16 | 14,03509 | 1.71E-07 | IL33, IL1RN, CXCL9, CXCL8, IL13, IFNA2, IL18, TNF, IL4, IL1A, CXCL10, IL6, IL5, IFNG, IL1B, IL17A | 88 | 298 | 8840 | 5,393533 | 4.03E-05 | 1.39E-06 | 8.72E-07 |
| KEGG_PATHWAY | hsa04151:PI3K-Akt signaling pathway | 17 | 14,91228 | 3.73E-07 | CDKN1B, FLT1, ITGB4, IFNA2, PIK3R1, EGFR, IGF1R, VEGFA, IL4, IL6, KITLG, SPP1, KDR, BCL2, TLR4, BCL2L1, TLR2 | 88 | 362 | 8840 | 4,717479 | 8.80E-05 | 2.93E-06 | 1.84E-06 |
| KEGG_PATHWAY | hsa04640:Hematopoietic cell lineage | 10 | 8,77193 | 4.78E-07 | IL4, IL1A, IL6, KITLG, IL5, IL1B, TNF, HLA-DRB1, HLA-DQA1, HLA-DQB1 | 88 | 99 | 8840 | 10,14692 | 1.13E-04 | 3.64E-06 | 2.28E-06 |
| KEGG_PATHWAY | hsa01524:Platinum drug resistance | 9 | 7,894737 | 6.26E-07 | GSTM1, CASP3, GSTP1, BCL2, XIAP, BIRC5, GSTT1, PIK3R1, BCL2L1 | 88 | 75 | 8840 | 12,05455 | 1.48E-04 | 4.62E-06 | 2.90E-06 |
| KEGG_PATHWAY | hsa05142:Chagas disease | 10 | 8,77193 | 6.71E-07 | IL6, JUN, CXCL8, IFNG, IL1B, PIK3R1, TNF, TLR4, MYD88, TLR2 | 88 | 103 | 8840 | 9,752868 | 1.58E-04 | 4.80E-06 | 3.01E-06 |
| KEGG_PATHWAY | hsa04064:NF-kappa B signaling pathway | 10 | 8,77193 | 7.91E-07 | VCAM1, CXCL8, PARP1, IL1B, BCL2, XIAP, TNF, TLR4, MYD88, BCL2L1 | 88 | 105 | 8840 | 9,5671 | 1.87E-04 | 5.49E-06 | 3.44E-06 |
| KEGG_PATHWAY | hsa05170:Human immunodeficiency virus 1 infection | 13 | 11,40351 | 1.02E-06 | JUN, IFNA2, HLA-B, TAP1, PIK3R1, TNF, CASP3, CFL1, BCL2, TLR4, MYD88, BCL2L1, TLR2 | 88 | 213 | 8840 | 6,131029 | 2.40E-04 | 6.86E-06 | 4.30E-06 |
| KEGG_PATHWAY | hsa05143:African trypanosomiasis | 7 | 6,140351 | 1.39E-06 | IL6, VCAM1, IFNG, IL1B, IL18, TNF, MYD88 | 88 | 37 | 8840 | 19,00491 | 3.28E-04 | 9.11E-06 | 5.71E-06 |
| KEGG_PATHWAY | hsa04658:Th1 and Th2 cell differentiation | 9 | 7,894737 | 3.02E-06 | IL4, JUN, IL5, IFNG, STAT1, IL13, HLA-DRB1, HLA-DQA1, HLA-DQB1 | 88 | 92 | 8840 | 9,827075 | 7.12E-04 | 1.92E-05 | 1.21E-05 |
| KEGG_PATHWAY | hsa05205:Proteoglycans in cancer | 12 | 10,52632 | 4.56E-06 | CASP3, MMP2, KDR, PIK3R1, TNF, TLR4, MMP9, DCN, EGFR, IGF1R, TLR2, VEGFA | 88 | 204 | 8840 | 5,909091 | 0.001075 | 2.83E-05 | 1.78E-05 |
| KEGG_PATHWAY | hsa04630:JAK-STAT signaling pathway | 11 | 9,649123 | 5.33E-06 | IL4, IL6, IL5, IFNG, STAT1, IFNA2, IL13, BCL2, PIK3R1, EGFR, BCL2L1 | 88 | 168 | 8840 | 6,577381 | 0.001257 | 3.23E-05 | 2.02E-05 |
| KEGG_PATHWAY | hsa05132:Salmonella infection | 13 | 11,40351 | 5.65E-06 | JUN, CXCL8, IL18, TNF, IL6, TUBB3, IL1B, CASP3, BCL2, NLRP3, TLR4, MYD88, TLR2 | 88 | 251 | 8840 | 5,202825 | 0.001334 | 3.34E-05 | 2.09E-05 |
| KEGG_PATHWAY | hsa05146:Amoebiasis | 9 | 7,894737 | 7.06E-06 | IL6, CXCL8, IFNG, IL1B, CASP3, PIK3R1, TNF, TLR4, TLR2 | 88 | 103 | 8840 | 8,777582 | 0.001665 | 4.06E-05 | 2.55E-05 |
| KEGG_PATHWAY | hsa05135:Yersinia infection | 10 | 8,77193 | 7.75E-06 | IL6, JUN, CXCL8, IL1B, IL18, NLRP3, PIK3R1, TNF, TLR4, MYD88 | 88 | 138 | 8840 | 7,279315 | 0.001828 | 4.36E-05 | 2.73E-05 |
| KEGG_PATHWAY | hsa05168:Herpes simplex virus 1 infection | 18 | 15,78947 | 1.03E-05 | STAT1, IFNA2, HLA-B, TAP1, PIK3R1, TNF, IL6, IFNG, IL1B, CASP3, BCL2, MYD88, HLA-DQA1, HLA-DRB1, TLR3, HLA-DQB1, BCL2L1, TLR2 | 88 | 522 | 8840 | 3,46395 | 0.002433 | 5.66E-05 | 3.55E-05 |
| KEGG_PATHWAY | hsa04936:Alcoholic liver disease | 10 | 8,77193 | 1.10E-05 | C4A, IL6, CXCL8, IL1B, CASP3, IFNA2, TNF, TLR4, MYD88, IL17A | 88 | 144 | 8840 | 6,97601 | 0.002583 | 5.88E-05 | 3.69E-05 |
| KEGG_PATHWAY | hsa05320:Autoimmune thyroid disease | 7 | 6,140351 | 1.21E-05 | IL4, IL5, IFNA2, HLA-B, HLA-DRB1, HLA-DQA1, HLA-DQB1 | 88 | 53 | 8840 | 13,26758 | 0.002851 | 6.35E-05 | 3.98E-05 |
| KEGG_PATHWAY | hsa01521:EGFR tyrosine kinase inhibitor resistance | 8 | 7,017544 | 1.24E-05 | IL6, BCL2, KDR, PIK3R1, EGFR, BCL2L1, IGF1R, VEGFA | 88 | 80 | 8840 | 10,04545 | 0.002933 | 6.38E-05 | 4.00E-05 |
| KEGG_PATHWAY | hsa04623:Cytosolic DNA-sensing pathway | 8 | 7,017544 | 1.59E-05 | IL33, CXCL10, IL6, IL1B, CASP3, IFNA2, IL18, NLRP3 | 88 | 83 | 8840 | 9,682366 | 0.003741 | 7.97E-05 | 5.00E-05 |
| KEGG_PATHWAY | hsa05202:Transcriptional misregulation in cancer | 11 | 9,649123 | 1.81E-05 | CCNA2, IL6, FLT1, CDKN1B, CXCL8, IGFBP3, MMP3, RARA, MMP9, BCL2L1, IGF1R | 88 | 193 | 8840 | 5,725389 | 0.004266 | 8.91E-05 | 5.59E-05 |
| KEGG_PATHWAY | hsa05165:Human papillomavirus infection | 14 | 12,2807 | 2.00E-05 | CDKN1B, ITGB4, STAT1, IFNA2, HLA-B, PIK3R1, TNF, EGFR, VEGFA, CCNA2, CASP3, IRF1, SPP1, TLR3 | 88 | 333 | 8840 | 4,223314 | 0.00472 | 9.66E-05 | 6.06E-05 |
| KEGG_PATHWAY | hsa05235:PD-L1 expression and PD-1 checkpoint pathway in cancer | 8 | 7,017544 | 2.70E-05 | JUN, IFNG, STAT1, PIK3R1, TLR4, EGFR, MYD88, TLR2 | 88 | 90 | 8840 | 8,929293 | 0.00636 | 1.27E-04 | 7.98E-05 |
| KEGG_PATHWAY | hsa05130:Pathogenic Escherichia coli infection | 11 | 9,649123 | 2.80E-05 | IL6, JUN, CXCL8, TUBB3, IL1B, CASP3, IL18, NLRP3, TNF, TLR4, MYD88 | 88 | 203 | 8840 | 5,44335 | 0.006597 | 1.27E-04 | 7.98E-05 |
| KEGG_PATHWAY | hsa04510:Focal adhesion | 11 | 9,649123 | 2.80E-05 | JUN, FLT1, ITGB4, BCL2, SPP1, KDR, XIAP, PIK3R1, EGFR, IGF1R, VEGFA | 88 | 203 | 8840 | 5,44335 | 0.006597 | 1.27E-04 | 7.98E-05 |
| KEGG_PATHWAY | hsa05131:Shigellosis | 12 | 10,52632 | 3.00E-05 | JUN, CXCL8, IL1B, BCL2, IL18, NLRP3, PIK3R1, TNF, TLR4, EGFR, MYD88, BCL2L1 | 88 | 249 | 8840 | 4,841183 | 0.007051 | 1.34E-04 | 8.37E-05 |
| KEGG_PATHWAY | hsa05215:Prostate cancer | 8 | 7,017544 | 4.70E-05 | CDKN1B, GSTP1, MMP3, BCL2, PIK3R1, MMP9, EGFR, IGF1R | 88 | 98 | 8840 | 8,200371 | 0.011027 | 2.05E-04 | 1.29E-04 |
| KEGG_PATHWAY | hsa01522:Endocrine resistance | 8 | 7,017544 | 5.02E-05 | JUN, CDKN1B, MMP2, BCL2, PIK3R1, MMP9, EGFR, IGF1R | 88 | 99 | 8840 | 8,117539 | 0.011768 | 2.15E-04 | 1.35E-04 |
| KEGG_PATHWAY | hsa04210:Apoptosis | 9 | 7,894737 | 5.37E-05 | JUN, PARP1, CASP3, BCL2, XIAP, BIRC5, PIK3R1, TNF, BCL2L1 | 88 | 136 | 8840 | 6,647727 | 0.012595 | 2.26E-04 | 1.42E-04 |
| KEGG_PATHWAY | hsa05166:Human T-cell leukemia virus 1 infection | 11 | 9,649123 | 6.24E-05 | CCNA2, IL6, JUN, HLA-B, XIAP, PIK3R1, TNF, HLA-DRB1, HLA-DQA1, BCL2L1, HLA-DQB1 | 88 | 223 | 8840 | 4,955157 | 0.014625 | 2.58E-04 | 1.62E-04 |
| KEGG_PATHWAY | hsa05163:Human cytomegalovirus infection | 11 | 9,649123 | 6.99E-05 | IL6, CXCL8, IL1B, CASP3, IFNA2, HLA-B, TAP1, PIK3R1, TNF, EGFR, VEGFA | 88 | 226 | 8840 | 4,889381 | 0.01635 | 2.84E-04 | 1.78E-04 |
| KEGG_PATHWAY | hsa04625:C-type lectin receptor signaling pathway | 8 | 7,017544 | 7.31E-05 | IL6, JUN, STAT1, IL1B, IRF1, NLRP3, PIK3R1, TNF | 88 | 105 | 8840 | 7,65368 | 0.01711 | 2.93E-04 | 1.83E-04 |
| KEGG_PATHWAY | hsa04672:Intestinal immune network for IgA production | 6 | 5,263158 | 1.12E-04 | IL4, IL6, IL5, HLA-DRB1, HLA-DQA1, HLA-DQB1 | 88 | 49 | 8840 | 12,30056 | 0.02598 | 4.39E-04 | 2.75E-04 |
| KEGG_PATHWAY | hsa05167:Kaposi sarcoma-associated herpesvirus infection | 10 | 8,77193 | 1.24E-04 | IL6, JUN, CXCL8, STAT1, CASP3, IFNA2, HLA-B, PIK3R1, TLR3, VEGFA | 88 | 196 | 8840 | 5,125232 | 0.028887 | 4.80E-04 | 3.01E-04 |
| KEGG_PATHWAY | hsa04612:Antigen processing and presentation | 7 | 6,140351 | 1.28E-04 | IFNG, HLA-B, TAP1, TNF, HLA-DRB1, HLA-DQA1, HLA-DQB1 | 88 | 80 | 8840 | 8,789773 | 0.029784 | 4.88E-04 | 3.06E-04 |
| KEGG_PATHWAY | hsa04010:MAPK signaling pathway | 12 | 10,52632 | 1.61E-04 | IL1A, JUN, KITLG, FLT1, IL1B, CASP3, KDR, TNF, EGFR, MYD88, IGF1R, VEGFA | 88 | 300 | 8840 | 4,018182 | 0.03726 | 5.93E-04 | 3.72E-04 |
| KEGG_PATHWAY | hsa05160:Hepatitis C | 9 | 7,894737 | 1.61E-04 | CXCL10, IFNG, STAT1, CASP3, IFNA2, PIK3R1, TNF, EGFR, TLR3 | 88 | 159 | 8840 | 5,686106 | 0.037273 | 5.93E-04 | 3.72E-04 |
| KEGG_PATHWAY | hsa05206:MicroRNAs in cancer | 12 | 10,52632 | 2.27E-04 | MIR16-1, CDKN1B, ABCB1, CASP3, MIR152, BCL2, HMOX1, PIK3R1, MMP9, EGFR, MIR125A, VEGFA | 88 | 312 | 8840 | 3,863636 | 0.052081 | 8.23E-04 | 5.16E-04 |
| KEGG_PATHWAY | hsa05207:Chemical carcinogenesis - receptor activation | 10 | 8,77193 | 2.49E-04 | JUN, GSTM1, UGT1A1, BCL2, XIAP, BIRC5, GSTT1, PIK3R1, EGFR, VEGFA | 88 | 215 | 8840 | 4,672304 | 0.057184 | 8.85E-04 | 5.55E-04 |
| KEGG_PATHWAY | hsa05225:Hepatocellular carcinoma | 9 | 7,894737 | 2.55E-04 | GSTM1, GSTP1, HMOX1, GSTT1, PIK3R1, ARID1A, EGFR, BCL2L1, IGF1R | 88 | 170 | 8840 | 5,318182 | 0.058359 | 8.85E-04 | 5.55E-04 |
| KEGG_PATHWAY | hsa04215:Apoptosis - multiple species | 5 | 4,385965 | 2.55E-04 | CASP3, BCL2, XIAP, BIRC5, BCL2L1 | 88 | 32 | 8840 | 15,69602 | 0.058417 | 8.85E-04 | 5.55E-04 |
| KEGG_PATHWAY | hsa05222:Small cell lung cancer | 7 | 6,140351 | 2.93E-04 | CDKN1B, CASP3, BCL2, RARB, XIAP, PIK3R1, BCL2L1 | 88 | 93 | 8840 | 7,561095 | 0.066925 | 0.001004 | 6.29E-04 |
| KEGG_PATHWAY | hsa04014:Ras signaling pathway | 10 | 8,77193 | 5.27E-04 | GRIN2A, KITLG, FLT1, KDR, PIK3R1, PLA2G6, EGFR, BCL2L1, IGF1R, VEGFA | 88 | 238 | 8840 | 4,220779 | 0.116938 | 0.001776 | 0.001114 |
| KEGG_PATHWAY | hsa05219:Bladder cancer | 5 | 4,385965 | 6.71E-04 | CXCL8, MMP2, MMP9, EGFR, VEGFA | 88 | 41 | 8840 | 12,25055 | 0.146578 | 0.002232 | 0.0014 |
| KEGG_PATHWAY | hsa04145:Phagosome | 8 | 7,017544 | 8.67E-04 | TUBB3, HLA-B, TAP1, TLR4, HLA-DRB1, HLA-DQA1, TLR2, HLA-DQB1 | 88 | 157 | 8840 | 5,118703 | 0.185133 | 0.002803 | 0.001758 |
| KEGG_PATHWAY | hsa04932:Non-alcoholic fatty liver disease | 8 | 7,017544 | 8.67E-04 | IL1A, IL6, JUN, CXCL8, IL1B, CASP3, PIK3R1, TNF | 88 | 157 | 8840 | 5,118703 | 0.185133 | 0.002803 | 0.001758 |
| KEGG_PATHWAY | hsa05210:Colorectal cancer | 6 | 5,263158 | 0.001613 | JUN, CASP3, BCL2, BIRC5, PIK3R1, EGFR | 88 | 87 | 8840 | 6,9279 | 0.316832 | 0.005145 | 0.003226 |
| KEGG_PATHWAY | hsa05208:Chemical carcinogenesis - reactive oxygen species | 9 | 7,894737 | 0.00166 | JUN, GSTM1, HMOX1, GSTT1, PIK3R1, SOD2, EGFR, SOD1, VEGFA | 88 | 226 | 8840 | 4,000402 | 0.32432 | 0.005223 | 0.003275 |
| KEGG_PATHWAY | hsa04915:Estrogen signaling pathway | 7 | 6,140351 | 0.002413 | JUN, MMP2, BCL2, RARA, PIK3R1, MMP9, EGFR | 88 | 139 | 8840 | 5,058862 | 0.434572 | 0.007396 | 0.004638 |
| KEGG_PATHWAY | hsa05322:Systemic lupus erythematosus | 7 | 6,140351 | 0.002413 | C4A, GRIN2A, IFNG, TNF, HLA-DRB1, HLA-DQA1, HLA-DQB1 | 88 | 139 | 8840 | 5,058862 | 0.434572 | 0.007396 | 0.004638 |
| KEGG_PATHWAY | hsa04380:Osteoclast differentiation | 7 | 6,140351 | 0.002686 | IL1A, JUN, IFNG, STAT1, IL1B, PIK3R1, TNF | 88 | 142 | 8840 | 4,951985 | 0.469903 | 0.008126 | 0.005096 |
| KEGG_PATHWAY | hsa04061:Viral protein interaction with cytokine and cytokine receptor | 6 | 5,263158 | 0.002975 | CXCL10, IL6, CXCL9, CXCL8, IL18, TNF | 88 | 100 | 8840 | 6,027273 | 0.505 | 0.008888 | 0.005574 |
| KEGG_PATHWAY | hsa01523:Antifolate resistance | 4 | 3,508772 | 0.003085 | IL6, IL1B, MTHFR, TNF | 88 | 30 | 8840 | 13,39394 | 0.517728 | 0.009102 | 0.005708 |
| KEGG_PATHWAY | hsa04218:Cellular senescence | 7 | 6,140351 | 0.004411 | CCNA2, IL1A, IL6, CXCL8, IGFBP3, HLA-B, PIK3R1 | 88 | 157 | 8840 | 4,478865 | 0.647675 | 0.012851 | 0.008059 |
| KEGG_PATHWAY | hsa05416:Viral myocarditis | 5 | 4,385965 | 0.004652 | CASP3, HLA-B, HLA-DRB1, HLA-DQA1, HLA-DQB1 | 88 | 69 | 8840 | 7,279315 | 0.667274 | 0.013227 | 0.008295 |
| KEGG_PATHWAY | hsa04664:Fc epsilon RI signaling pathway | 5 | 4,385965 | 0.004652 | IL4, IL5, IL13, PIK3R1, TNF | 88 | 69 | 8840 | 7,279315 | 0.667274 | 0.013227 | 0.008295 |
| KEGG_PATHWAY | hsa04015:Rap1 signaling pathway | 8 | 7,017544 | 0.004766 | GRIN2A, KITLG, FLT1, KDR, PIK3R1, EGFR, IGF1R, VEGFA | 88 | 212 | 8840 | 3,790738 | 0.676158 | 0.013391 | 0.008398 |
| KEGG_PATHWAY | hsa05020:Prion disease | 9 | 7,894737 | 0.005895 | IL1A, IL6, GRIN2A, TUBB3, IL1B, CASP3, PIK3R1, TNF, SOD1 | 88 | 278 | 8840 | 3,252126 | 0.752266 | 0.016368 | 0.010265 |
| KEGG_PATHWAY | hsa05212:Pancreatic cancer | 5 | 4,385965 | 0.006863 | STAT1, PIK3R1, EGFR, BCL2L1, VEGFA | 88 | 77 | 8840 | 6,523022 | 0.803123 | 0.018783 | 0.011779 |
| KEGG_PATHWAY | hsa04660:T cell receptor signaling pathway | 6 | 5,263158 | 0.006924 | IL4, JUN, IL5, IFNG, PIK3R1, TNF | 88 | 122 | 8840 | 4,940387 | 0.805978 | 0.018783 | 0.011779 |
| KEGG_PATHWAY | hsa00983:Drug metabolism - other enzymes | 5 | 4,385965 | 0.008191 | GSTM1, UGT1A1, GSTP1, GSTT1, XDH | 88 | 81 | 8840 | 6,200898 | 0.856441 | 0.021966 | 0.013776 |
| KEGG_PATHWAY | hsa04926:Relaxin signaling pathway | 6 | 5,263158 | 0.008996 | JUN, MMP2, PIK3R1, MMP9, EGFR, VEGFA | 88 | 130 | 8840 | 4,636364 | 0.881482 | 0.023855 | 0.01496 |
| KEGG_PATHWAY | hsa04068:FoxO signaling pathway | 6 | 5,263158 | 0.009574 | IL6, CDKN1B, PIK3R1, SOD2, EGFR, IGF1R | 88 | 132 | 8840 | 4,566116 | 0.896735 | 0.025106 | 0.015745 |
| KEGG_PATHWAY | hsa05226:Gastric cancer | 6 | 5,263158 | 0.015971 | CDKN1B, ABCB1, BCL2, RARB, PIK3R1, EGFR | 88 | 150 | 8840 | 4,018182 | 0.977622 | 0.04142 | 0.025975 |
| KEGG_PATHWAY | hsa05022:Pathways of neurodegeneration - multiple diseases | 11 | 9,649123 | 0.019786 | IL1A, ATXN3, IL6, GRIN2A, TUBB3, IL1B, CASP3, BCL2, TNF, BCL2L1, SOD1 | 88 | 483 | 8840 | 2,287785 | 0.991051 | 0.050755 | 0.031829 |
| KEGG_PATHWAY | hsa04213:Longevity regulating pathway - multiple species | 4 | 3,508772 | 0.022951 | PIK3R1, SOD2, SOD1, IGF1R | 88 | 62 | 8840 | 6,480938 | 0.995828 | 0.05824 | 0.036523 |
| KEGG_PATHWAY | hsa05120:Epithelial cell signaling in Helicobacter pylori infection | 4 | 3,508772 | 0.032576 | JUN, CXCL8, CASP3, EGFR | 88 | 71 | 8840 | 5,659411 | 0.999597 | 0.080926 | 0.05075 |
| KEGG_PATHWAY | hsa05204:Chemical carcinogenesis - DNA adducts | 4 | 3,508772 | 0.032576 | GSTM1, UGT1A1, GSTP1, GSTT1 | 88 | 71 | 8840 | 5,659411 | 0.999597 | 0.080926 | 0.05075 |
| KEGG_PATHWAY | hsa04622:RIG-I-like receptor signaling pathway | 4 | 3,508772 | 0.033757 | CXCL10, CXCL8, IFNA2, TNF | 88 | 72 | 8840 | 5,580808 | 0.999698 | 0.082986 | 0.052042 |
| KEGG_PATHWAY | hsa00982:Drug metabolism - cytochrome P450 | 4 | 3,508772 | 0.03496 | GSTM1, UGT1A1, GSTP1, GSTT1 | 88 | 73 | 8840 | 5,504359 | 0.999775 | 0.085058 | 0.053341 |
| KEGG_PATHWAY | hsa04115:p53 signaling pathway | 4 | 3,508772 | 0.037432 | CASP3, IGFBP3, BCL2, BCL2L1 | 88 | 75 | 8840 | 5,357576 | 0.999877 | 0.090143 | 0.056531 |
| KEGG_PATHWAY | hsa04650:Natural killer cell mediated cytotoxicity | 5 | 4,385965 | 0.039014 | IFNG, CASP3, IFNA2, PIK3R1, TNF | 88 | 130 | 8840 | 3,863636 | 0.999917 | 0.093004 | 0.058325 |
| KEGG_PATHWAY | hsa00980:Metabolism of xenobiotics by cytochrome P450 | 4 | 3,508772 | 0.042639 | GSTM1, UGT1A1, GSTP1, GSTT1 | 88 | 79 | 8840 | 5,086306 | 0.999966 | 0.100627 | 0.063105 |
| KEGG_PATHWAY | hsa05203:Viral carcinogenesis | 6 | 5,263158 | 0.051224 | CCNA2, JUN, CDKN1B, CASP3, HLA-B, PIK3R1 | 88 | 205 | 8840 | 2,940133 | 0.999996 | 0.119692 | 0.075061 |
| KEGG_PATHWAY | hsa04012:ErbB signaling pathway | 4 | 3,508772 | 0.052574 | JUN, CDKN1B, PIK3R1, EGFR | 88 | 86 | 8840 | 4,672304 | 0.999997 | 0.121642 | 0.076284 |
| KEGG_PATHWAY | hsa04514:Cell adhesion molecules | 5 | 4,385965 | 0.068774 | VCAM1, HLA-B, HLA-DRB1, HLA-DQA1, HLA-DQB1 | 88 | 157 | 8840 | 3,199189 | 1 | 0.157579 | 0.098821 |
| KEGG_PATHWAY | hsa04750:Inflammatory mediator regulation of TRP channels | 4 | 3,508772 | 0.073684 | IL1B, PIK3R1, TRPM8, PLA2G6 | 88 | 99 | 8840 | 4,05877 | 1 | 0.167205 | 0.104858 |
| KEGG_PATHWAY | hsa05150:Staphylococcus aureus infection | 4 | 3,508772 | 0.075444 | C4A, HLA-DRB1, HLA-DQA1, HLA-DQB1 | 88 | 100 | 8840 | 4,018182 | 1 | 0.169569 | 0.10634 |
| KEGG_PATHWAY | hsa05010:Alzheimer disease | 8 | 7,017544 | 0.089724 | IL1A, IL6, GRIN2A, TUBB3, IL1B, CASP3, PIK3R1, TNF | 88 | 391 | 8840 | 2,055336 | 1 | 0.199762 | 0.125274 |

**Figure S1** Cellular Component (CC) Enrichment analysis.


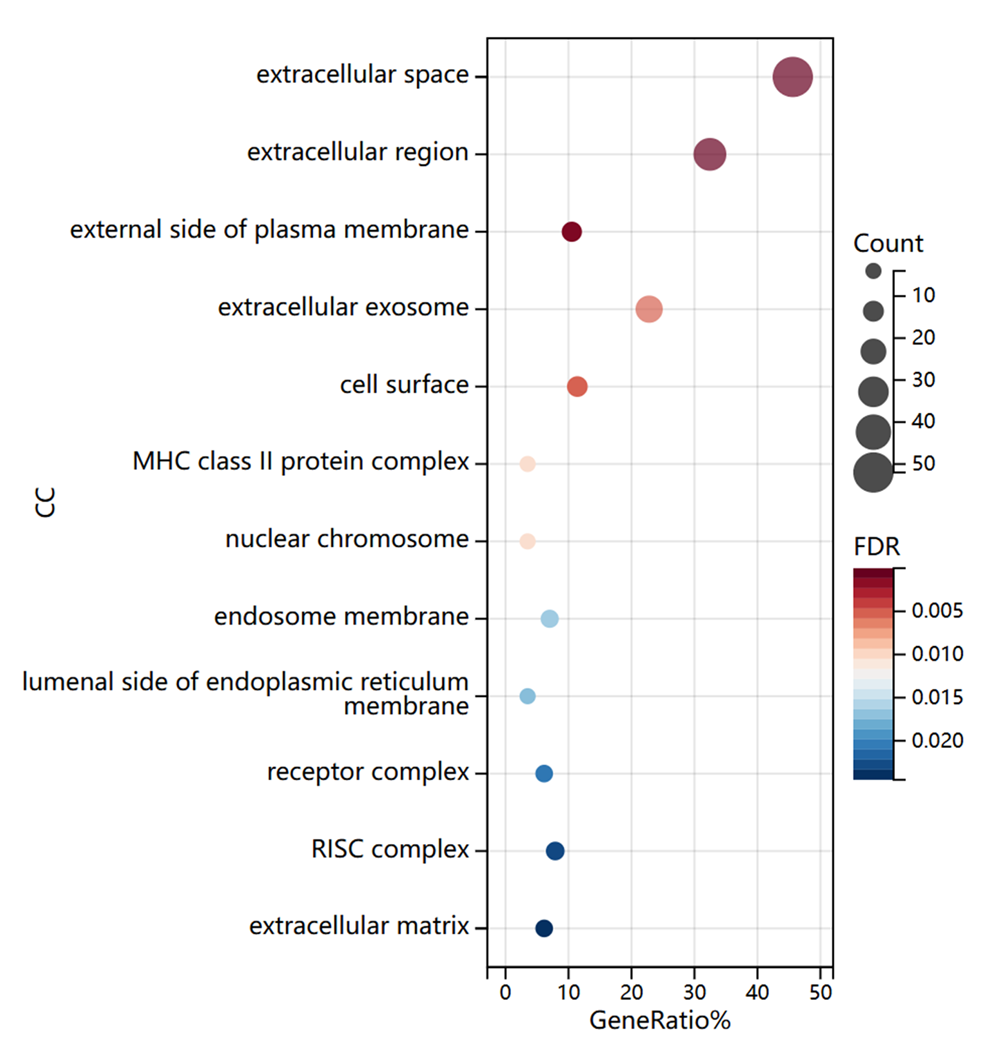

Supplement: Supplementary file 1 [file mmc1.docx]
